# Impact of Additives on Poly(acrylonitrile-butadiene-styrene) Membrane Formation Process Using Non-Solvent-Induced Phase Separation

**DOI:** 10.3390/membranes15060181

**Published:** 2025-06-16

**Authors:** Sulaiman Dhameri, Jason Stallings, Endras Fadhilah, Emily Ingram, Mara Leach, Anastasiia Aronova, Malgorzata Chwatko

**Affiliations:** Chemical and Materials Engineering Department, Pigman College of Engineering, University of Kentucky, Lexington, KY 40506, USA

**Keywords:** ABS, non-solvent-induced phase separation

## Abstract

Poly(acrylonitrile-butadiene-styrene) (ABS) is a common polymer used in toys, automobile parts, and membranes. Membranes fabricated with this copolymer commonly employ toxic solvents and have a dense architecture, which may not work in all applications. This work investigates the synthesis of ABS membranes, using green solvents and the influence of additives on the phase inversion process during the non-solvent-induced phase separation. The addition of water-soluble additives, ethanol, and acetone is hypothesized to provide additional control over viscosity and volatility, and, consequently, impact the phase inversion process. Membranes were fabricated with PolarClean and with various additive concentrations and evaporation times. The resulting membranes were characterized using scanning electron microscopy (SEM) and a pycnometer to visualize the pore structure and obtain porosity information. Membrane performance, including water flux and bovine serum albumin rejection, was evaluated using dead-end cell filtration. Membranes fabricated using only PolarClean had fingerlike pore morphology and relatively low protein rejection. The addition of additives resulted in a change in pore architecture and rejection, which is hypothesized to be a result of additives’ volatility, humidity, and destabilization of liquid–liquid separation. This study provides a more detailed understanding of the impact of additives on the resulting ABS membrane structure and performance, with a focus on safer solvents.

## 1. Introduction

Membrane technology is widely used in many industrial applications, such as virus or protein separation in aqueous solutions [1,2]. Depending on the solution chemistry, different membrane materials may be necessary to perform the desired separation, maintain competitive cost, and ensure long-term stability. Poly(acrylonitrile-butadiene-styrene) (ABS) is known for its cost-effectiveness, balanced mechanical properties, and moderate glass transition temperature of 110 °C [3,4]. It combines hydrophobic poly(butadiene) domains suspended in a matrix of poly(acrylonitrile-styrene) to provide increased strength, rigidity, and toughness. Due to these favorable properties, ABS has been used to create membranes for applications such as gas separation, air filtration, dewatering of algae, and particle filtration [5,6,7,8,9,10].

There are a few techniques to choose from when creating porous flat sheet membranes. One of the most employed methods for the creation of these membranes is non-solvent-induced phase separation (NIPS). The process is thermodynamically driven to precipitate the polymer as its solubility quickly decreases in the presence of a non-solvent, typically water. When this process involves amphiphilic copolymers, there are also significant kinetic considerations, as polyacrylonitrile is more polar and may lead to greater solubility in water versus polybutadiene. Generally, this behavior has proven difficult to form ABS membranes using water as a non-solvent [11].

ABS membrane fabrication traditionally has relied on the use of toxic solvents, which pose significant threats to the environment and human health [12,13,14]. For example, Kamelian et al. used N-Methyl-2-pyrrolidone, dimethylacetamide, and hexane to fabricate ABS membranes, which are known to be toxic solvents for humans and the environment [11]. Due to the growing changes in regulations, such as the European Union’s REACH Regulation and the United States’ n-Methyl pyrrolidone Regulation Under the Toxic Substances Control Act (TSCA), many industries are transitioning towards using green solvents [15]. The use of green solvents promotes a more sustainable approach to membrane production, focusing on both the safety of the solvents to individuals, the environment, and the cradle-to-grade considerations of manufacturing [16,17,18]. Many recent publications note the possibility of using water-soluble green solvents, such as PolarClean^®^, gamma-valerolactone, and dimethyl sulfoxide to produce polymeric membranes, which can be used as drop in replacements in membrane formation processes, such as non-solvent-induced phase separation; however, to our knowledge, this has not been performed to create ABS membranes [19,20,21].

In addition to a primary solvent, additives are also used in membrane fabrication to tune the phase separation process. These secondary additives can serve many purposes, such as viscosity or volatility modifiers, and prior research has shown the importance of both factors in the phase inversion process. Solution viscosity impacts the speed of the demixing process, with lower-viscosity solutions leading to more porous structures with larger pores [22,23]. Solvent volatility, on the other hand, is closely tied to the surface layer’s porosity and pore size. Numerous studies have shown the impact of volatility and evaporation on the surface’s densification, which in turn impacts permeability and rejection of the membranes to different contaminants [24,25].

In this work, we aim to fabricate ABS membranes using green solvents and to study the impacts of additives on the phase inversion process. Specifically, we examined the effects of acetone and ethanol, which were selected as additives due to their higher volatility and lower viscosity than PolarClean. These solvents are generally greener than commonly used solvents in membrane fabrication and can be widely found in many consumer products [26,27,28]. To understand the additives’ effects on the phase separation process, we investigated how the solubility and viscosity of ABS were altered using Hansen solubility parameters and viscosity measurements. We also determined the physical properties of the membranes, such as hydrophobicity, porosity, and pore structure. The detailed characterization allowed us to determine the changes in the phase inversion process during the addition of the additives, which in turn increased protein rejection of the membranes.

## 2. Materials and Methods

### 2.1. Chemical Materials

ABS used in this study was purchased from McMaster-Carr (Santa Fe Springs, CA, USA). Rhodiasolv^®^ PolarClean was kindly provided by Solvay Novecare (Princeton, NJ, USA). Reagent ethanol and acetone were purchased from Fisher Scientific (Waltham, MA, USA) and VWR (Radnor, PA, USA). Bovine serum albumin (BSA) with an average molecular weight of 66,463 Da was purchased from Fisher Scientific. Type I deionized water was obtained from a Millipore Direct-Q^®^ 3 UV Water Purification System.

### 2.2. ABS Characterization

^1^H NMR spectroscopy was carried out on a 500 MHz JEOL ECZr spectrometer (Peabody, MA, USA). Spectra were collected in deuterated chloroform at approximately 5 mg/mL concentration. Differential scanning calorimetry (DSC) was performed using TA Instruments DSC250 (New Castle, DE, USA). The temperature ranged from −90 to +150 °C and was controlled by heating and cooling at 10 °C/min. Size exclusion chromatography (SEC) was carried out on an Agilent system (Santa Clara, CA, USA) with a 1260 infinity 2 isocratic pump, degasser, and temperature-controlled column chamber held at 30 °C containing Agilent PLgel 5 μm MIXED-C columns with an operating range of 500–10,000,000 g/mol relative to polystyrene standards. Ethyl acetate was used as the mobile phase at 1 mL/min. A suite of detectors from Wyatt Technologies (Goleta, CA, USA) provided measurements of polymer concentration and molecular weight. Static light scattering was measured using a DAWN 8 Multi-Angle static Light Scattering system, and the differential refractive index was measured with an Optilab TrEX.

### 2.3. CHEM 21 Solvent Evaluation

The solvent was evaluated according to the following three categories: safety score, environmental score, and health score. The scores were determined by looking at the solvent’s safety data sheet and identifying data on the flash point, boiling point, and different hazards. The publication by Prat et al. outlines the scores attributed to different features of the solvent, which were used in this publication [29].

### 2.4. Dope Solution Creation

The solutions were prepared by combining ABS, PolarClean, and a cosolvent in varying ratios within glass vials. The vials were heated to 40 °C and stirred at 250 RPM for a maximum of four days to allow for the full dissolution of the polymer. The polymeric solutions were formulated with ABS at a concentration of 16 wt%.

### 2.5. Solubility of ABS

The degree of affinity between the solvent and polymer was assessed through Hansen solubility parameters (HSPs). The calculation is expressed as follows:

(1) $$\delta^{2}=\delta_{d}^{2}+\delta_{p}^{2}+\delta_{h}^{2}$$

Here, *δ* represents the total solubility parameter (in MPa^0.5^), *δ_d_* corresponds to the dispersion cohesion solubility parameter (in MPa^0.5^), *δ_p_* denotes the polar cohesion solubility parameter (in MPa^0.5^), and *δ_h_* refers to the hydrogen-bonding cohesion parameter (in MPa^0.5^). To gauge the compatibility between two molecules, the following expression was used:

(2) $$R_{a}=\sqrt{(\delta_{ds}-\delta_{dp}{)}^{2}+(\delta_{ps}-\delta_{pp}{)}^{2}+(\delta_{hs}-\delta_{hp}{)}^{2}}$$

In this equation, *R_a_* represents the HSP distance (in MPa^0.5^), with *δ_ds_*, *δ_ps_*, and *δ_hs_* representing the partial solubility parameters for the solvent (in MPa^0.5^), while *δ_dp_*, *δ_pp_*, and *δ_hp_* correspond to the partial solubility parameters for the polymer (in MPa^0.5^). The parameters are provided in Table S2 in the Supplementary Materials [30,31,32,33,34,35].

For a more comprehensive understanding of the relative energy difference (*RED*) the following equation was used, where *R_o_* is the radius of the Hansen solubility parameter sphere for the polymer (*R_o_* of the ABS is 9.4 Mpa^0.5^):

(3) $$RED=R_{a}/R_{o}$$

### 2.6. Volatility of Dope Solution

To evaluate the volatility of casting solutions, 3 mL of each solvent and their blends were placed on a glass Petri dish and its weight change was measured over time using an analytical balance (Scout™ Pro 200 g, Ohaus, Parsippany, NJ, USA). The experiment was performed for ten minutes at room temperature in a fume hood with moderate airflow velocity, matching the experimental conditions during membrane casting. Volatility was calculated as the weight changed over time.

### 2.7. Viscosity of Dope Solution

After thoroughly mixing the dope solutions, viscosity was measured using a Rheometer Discover HR-2 (TA instrument, DE) equipped with a 40 mm flat plate. The temperature was kept at 25 °C, while the shear rate varied from 0 to 80 s^−1^. The viscosity of solutions with 5 wt% water was measured approximately five minutes after adding water to the previously dissolved samples. Any precipitate that formed was not included in the sample taken for viscosity measurement.

### 2.8. Membrane Fabrication

The membranes were cast at room temperature in the fume hood with moderate air flow. Approximately 5 mL of dope solution was poured onto a glass plate and quickly cast using a stainless steel doctor blade set to 0.035 inch. Then, the plate was immersed in a coagulation water bath at room temperature. For membranes with additional evaporation time, the cast film was left in a fume hood for a set amount of time (0, 0.5, or 1 min) prior to water bath immersion. All membranes were left in the water bath from distilled water for at least 2 min and transferred to a secondary container full of distilled water for over twenty-four hours. This was performed to complete the phase inversion and to remove leftover solvents prior to other tests.

### 2.9. SEM

The film’s morphology was analyzed using scanning electron microscopy (SEM). Membrane samples were first frozen by submersion in liquid nitrogen and fractured afterward. The samples were sputter-coated (Leica ACE 600 carbon) with 2 nm of palladium to enhance the sample’s conductivity prior to imaging. The cross-sections were imaged on a Quanta FEG-250 SEM. The top-section was imaged on FEI Helios Nanolab 660 SEM. The size of the top surface pores was determined by image analysis, using MATLAB (version R2024b) and ImageJ (version 1.54g). MATLAB script has been published and validated against manual measurements [36]. Three images were used for analysis. Sample containing 20 wt% acetone was not successfully analyzed by the pore script and was instead manually analyzed with ImageJ. Manual analysis revealed that the smallest pores were 6 nm; therefore, a cut-off of 5 nm was used in the script.

### 2.10. Porosity

Porosity was determined using an Ultrapyc 500 pycnometer from Anton Parr (Vernon Hills, IL, USA). Helium was used as the test gas at 5 psig. The pycnometer provided the true volume of the sample, while the geometric volume was determined using a ruler and micrometer. The *porosity* was determined using the following equation:

(4) $$Porosity=1-\frac{Volume_{true}}{Volume_{geometric}}\times 100$$

### 2.11. Mechanical Characterization

For mechanical characterization, rectangular specimens 50 mm in length and 10 mm wide were tested under tension using a 5 kN load cell on a Instron universal testing machine (Model 3345). The tests were conducted at ambient conditions and at a 5 mm/min crosshead speed until failure. Force and displacement data were recorded continuously throughout the test to determine key tensile properties, including tensile strength and elongation at break.

### 2.12. Water Contact Angle

The contact angle was measured using a drop-shape analyzer connected to a high-definition camera (DSA 100S, Kruss Company, Hamburg, Germany). To measure the contact angle, a drop containing 2 µL of deionized water was deposited on the membrane surface, and the camera captured the interface between the water droplet and the membrane surface. Contact angles were measured for 40 s for each sample and were further used to calculate the mean and standard deviation.

### 2.13. Water Flux and BSA Rejection

An Amicon dead-end filtration cell (Amicon Stirred Cell UFSC20001) was used to test water permeation under a constant pressure of 20 psi at room temperature. The pure water flux was measured by filtering deionized water through the membranes. Next, solutions containing 100 mg/L BSA were filtered through the same membranes under the same pressure and room temperature. Throughout the filtration process, permeability was recorded, and permeate samples were collected to analyze the concentrations of BSA in both the feed and permeate using a microplate reader (Biotek Synergy H1, Winooski, VT, USA). BSA concentration was compared to a calibration curve collected at known concentrations of BSA at a wavelength of 280 nm.

## 3. Results and Discussion

### 3.1. Polymer Characterization

**Figure 1:**
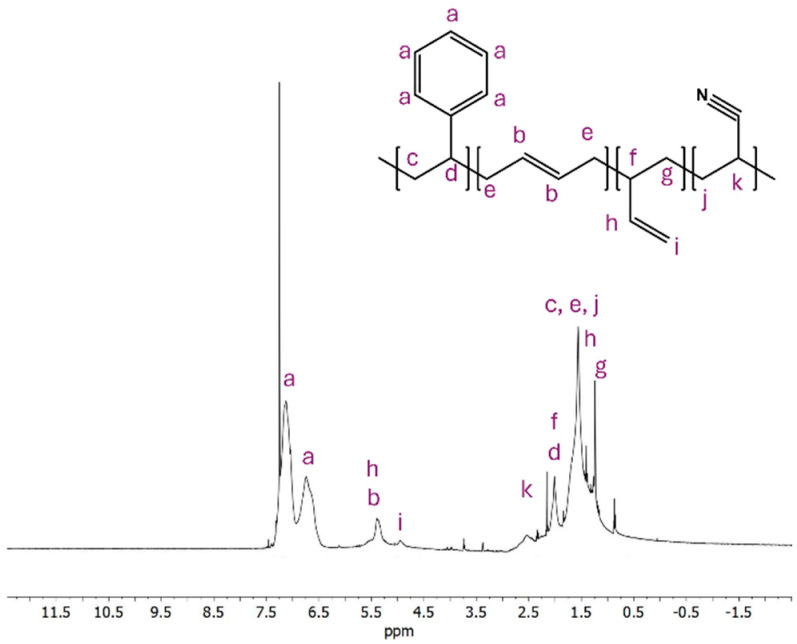
ABS ^1^H NMR spectra showing a composition of 48% poly(styrene), 14% poly(butadiene), 38% poly(acrylonitrile) performed in CDCl_3_.

Prior to membrane synthesis, polymer properties impacting solution viscosity and phase inversion were evaluated. The ratio of each monomer was determined using ^1^H NMR spectroscopy as shown in Figure 1. By analyzing the designated peaks, we found that ABS contained approximately 48% polystyrene, 14% polybutadiene, and 38% polyacrylonitrile. Using differential scanning calorimetry (DSC), the glass transition temperature was found to be 101.825 °C, as shown in the Supplementary Materials (Figure S13). This transition captures the poly(acrylonitrile)-poly(styrene) domain. Poly(butadinene) has a glass transition lower than the range of the instrument used in the study, and as such was not captured.

In addition to the composition, molecular weight is an important parameter that impacts polymer phase inversion. Size exclusion chromatography was used to determine molecular weight, and the resulting plot can be found in Supplementary Materials (Figure S14). Using conventional column calibration, the molecular weight of ABS was determined to be 80.05 kg/mol with PDI of 1.645. Since the calibration curve was built using linear polystyrene, most likely, the calibration did not perfectly replicate the behavior of ABS as it traveled through the column and, as such, might underestimate the true molecular weight of the polymer.

### 3.2. Solvent Selection

The study’s first goal is to identify a suitable green solvent for ABS. Hansen’s solubility parameters were used to estimate the relative energy differences in various common green solvents, such as PolarClean, γ-valerolactone, and Cyrene, all of which are miscible with water. This analysis suggested that among our selected solvents, only PolarClean possessed favorable relative energy differences, as seen in Supplementary Materials Table S1. According to the manufacturer’s (Solvay Novecare) specifications, Rhodiasolv^®^ PolarClean provides an excellent eco-friendly and safety profile. To confirm this, we have performed a green chemistry safety analysis using the CHEM21 selection guide [29]. The CHEM21 method allows for an evaluation of the solvent’s safety, health, and environmental score to determine its overall rating as recommended, problematic, or hazardous. As noted in Table 1, PolarClean had favorable scores (<3) in the safety, health, and environmental impact categories.

As aforementioned, solvent viscosity and volatility are also critical parameters that are directly linked to the pore formation process. PolarClean has relatively high viscosity and low volatility, which led us to consider the addition of additives to allow for more control over these properties. Ethanol and acetone can serve as good additives due to their CHEM21 selection guide recommended categories. While both solvents have high volatility, which may lead to potential high VOCs in a manufacturing setting, at low concentrations, the additives may help in the formation of a membrane with a dense top layer, which often is responsible for the high selectivity of the membrane against a contaminant.

Before adding the solvents to the ABS solution with PolarClean, it was necessary to assess the impact of the additives on the solubility of ABS. Table 2 shows the viscosities and calculated RED of 16 wt% ABS solutions with different additives’ compositions. The RED remained relatively constant, regardless of the addition of the additives, meaning that the solution remained a good solvent system overall. The addition of both ethanol and acetone led to a significant drop in viscosity compared to a pure PolarClean single solvent system. This drop in viscosity is anticipated to yield more porous membranes. Moreover, we also wanted to determine if the volatility of the solutions changed with the addition of additives to tune the membrane surface. To do this, we analyzed the weight change in the solutions over time to estimate the volatility of the solutions. Table 2 shows that the addition of cosolvents resulted in an increase in solution volatility. We suspect that only the additives are evaporating during this period, rather than PolarClean due to its low volatility, but this feature is still expected to aid in densifying the membrane surface during phase inversion. Lastly, it is worth noting that 10 wt% acetone and 20 wt% ethanol solutions have comparable viscosity and volatility characteristics, and is expected to form similar membrane structures [38,39].

### 3.3. Impact of Additives on Membrane Formation

**Figure 2:**
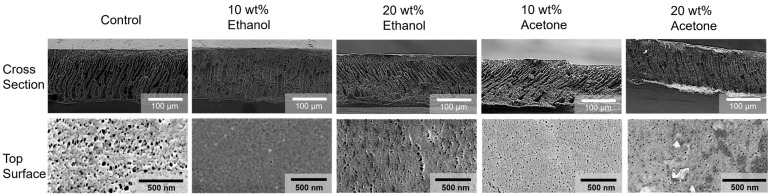
SEM images of membranes’ surfaces and cross-sections after fracturing in liquid nitrogen.

ABS membranes were fabricated with pure PolarClean and mixtures of additives using established methodology to evaluate the impact of solvent properties on the membrane formation [30,40]. The resulting membranes were analyzed using SEM, as shown in Figure 2. The ABS membranes using PolarClean showed finger-like pores that spanned the full length of the membrane. The ethanol-containing fabricated membranes showed similar finger-like morphology; however, there is a transition to the sponge-like structure noted by a lower number of pores spanning the whole length of the membrane. Acetone-containing membranes generally also had similar finger-like pore structure spanning the membrane, with a more sponge-like bottom layer of the membrane. Finger-like morphology of these membranes may be correlated with the solubility behavior of ABS. Due to the poly(butadiene) domain, ABS is suspended in a polymer solution, acting more as individual micelles than dissolved polymer chains. Due to the poor solubility of poly(butadiene) and the relatively low polystyrene and polyacrylonitrile concentration in solution, the polymer can precipitate quickly and hinder the entanglement of the poly(acrylonitrile)–poly(styrene) chains. This allows the non-solvent to diffuse into the cast film faster and form a finger-like structure [41]. When the viscosity and volatility are matched, comparing 20 wt% ethanol and 10 wt% acetone membranes, a similar mixed morphology is shown with some characteristics of a sponge-like and finger-like architecture. The top surface changes in pore diameter size may be due to the volatility of the solvents, allowing for densification of the surface and a more rapid precipitation.

**Figure 3:**
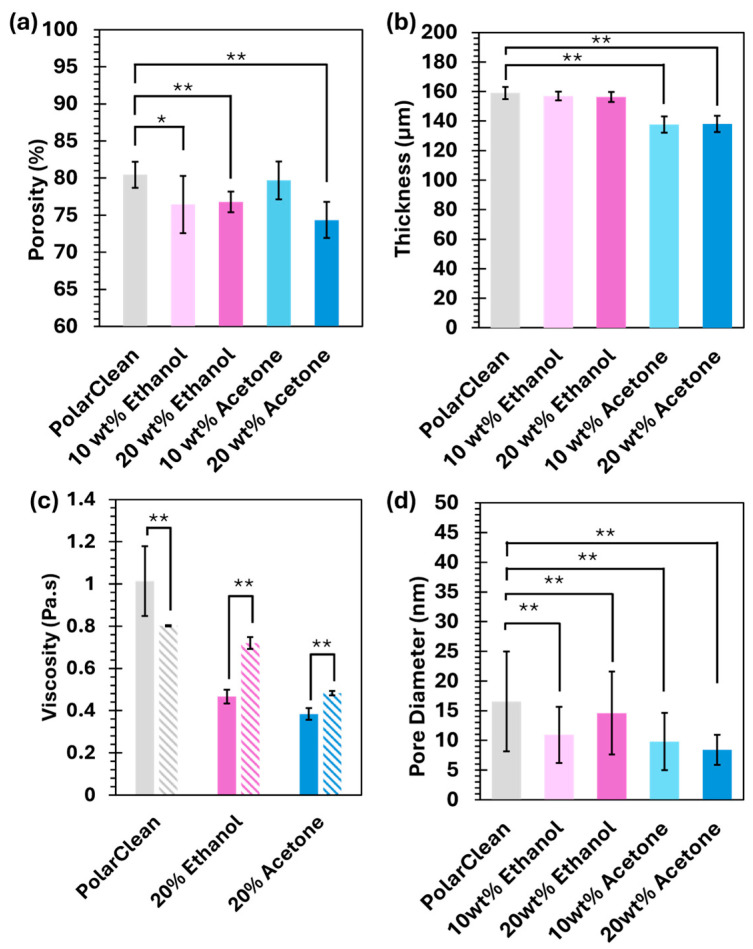
Effect of additives on the (**a**) porosity and (**b**) thickness of PolarClean of 16 wt% ABS membranes. (**c**) Impact of water on solution phase viscosity, where solid bars note solutions without water and striped bars note solutions containing 100 µL of water. (**d**) Pore size of the membranes’ top surface. All data are presented as mean ± s.d. independent samples involved (**a**) porosity, *n* = 3, (**b**) thickness, *n* = 1 with 4 technical replicates, (**c**) viscosity, *n* ≥ 2, and (**d**) pore diameter, *n* = 1 with 3 technical replicates. Two-tailed Student’s *t*-test analyzed statistical significance in both (**a**,**b**) compared to the pure sample, where * *p* < 0.05, ** *p* < 0.01.

To further explore the membrane structure and determine the cause of the microstructure change, we determined the porosity and thickness of the membranes. As shown in Figure 3, PolarClean membranes had a high porosity, which was significantly decreased in other formulations except for 10 wt% acetone. The decrease in porosity aligns with the transition to a more sponge-like structure noted in the SEM images. For the 10 wt% acetone membrane, the membrane retained its porosity due to some volatilization of the acetone but the initiation of a nucleation and growth mechanism to create larger pores still occurred [41,42]. The 20% acetone had much higher volatility ahead of phase inversion and prevented the same growth as in 10% acetone membranes. In addition to the pore morphology, we also analyzed the thickness of the membranes to see if the different formulations led to varying levels of structure collapse during the phase inversion process. Notably, acetone-containing solutions led to significantly thinner membranes than when pure PolarClean, or ethanol additive solutions were used. This decrease in membrane thickness may indicate that a moderate amount of evaporation is occurring before phase inversion. However, since the evaporation time was negligible, we do not believe that the changes in microstructure are only a result of additive evaporation, as ethanol samples also presented a change and are less volatile. A more likely explanation is that the additives altered the demixing kinetics, leading to a faster solidification of the finger-like pores.

### 3.4. Mixture Properties Affecting Membrane Structure

To achieve a better understanding of the factors that impacted the resulting substructure, additional factors were identified from the literature. The formation of the sponge-like layer is commonly attributed to a slower demixing or poorer solvent miscibility with the non-solvent [43,44]. However, in this study, a shift from finger-like to more sponge-like morphology is likely not due to a generally slower demixing since all solutions were more viscous than those using traditional solvents, and as such, all would be slower. Instead, the changes in the resulting substructure are likely related to the solubility and miscibility of water in the casting solutions. While the solution stability is typically assessed via a ternary phase diagram, ABS solutions were too opaque to determine cloud points due to the poor solubility of polybutadiene, which is a common problem faced in the fabrication of ABS membranes. Nonetheless, rapid polymer precipitation at even 5 wt% of water was observed. This rapid precipitation is likely attributed to the butadiene domain, which is not soluble in polar solvents. Interestingly, the ethanol-containing solutions were able to re-suspend the polymer in the solvent over a span of 24 h after water addition. Thus, the precipitation rate of ABS may mostly depend on the solubility of the polystyrene and polyacrylonitrile blocks.

Viscosity analysis was used to track the impact of water addition on the ABS solutions to evaluate changes in the polymer’s solubility. The results in Figure 3 show that, in the pure PolarClean solution, a notable decrease in viscosity was observed following the addition of water, suggesting a simple dilution effect without significant interactions between the solvents and polymer chains. This observation makes sense as polyacrylonitrile is less likely to hydrogen bond with water versus maintaining dipole–dipole interactions between the nitrile groups. The viscosity decrease from the addition of water further supports the more rapid diffusion of water into the cast film, favoring the formation of finger-like pores before complete polymer precipitation. When acetone is present in the starting solution, there is a mild increase in viscosity, which may indicate some interactions between water and acetone. These interactions can include hydrogen bonding, which has been studied extensively between water and acetone [45,46]. However, these are not sufficient to cause a significant impact on the phase inversion process to proceed via a spinodal decomposition. In the case of ethanol, there is a significant increase in viscosity upon the addition of water. This is indicative of more significant interactions between ethanol and water or ethanol and the polymers. Such significant changes in viscosity are often noted as indicators of liquid–liquid or upper critical solution temperature (UCST) in other studies [47].

To confirm the UCST presence for the ethanol-containing solutions, the solubility at different temperatures was evaluated. We found that the ABS ethanol PolarClean solutions containing 5 wt% of water had a thermal transition at 50 °C from a soluble to an insoluble mixture, as shown in the Supplemental Material (Figure S12). This temperature-based response suggests the presence of upper critical solution temperature behavior, confirming the viscosity results. Interestingly, in different polymer-solvent combinations used in this study, the solidification behavior was not displayed as observed in the ABS, PolarClean, ethanol, and water systems. UCST transitions have long been correlated with intermolecular interactions triggering phase separation [48,49]. At these low volume fractions of water, water molecules exist as free molecules, competing for hydrogen bonding with PolarClean and ethanol [50]. These intermolecular interactions also lower ABS solubility, as noted by the Hansen solubility coefficient in Supplementary Materials (Table S2). All together, these findings suggest the existence of a significant liquid–liquid separation as a driver in the formation of ethanol-containing membranes, which is different from the pure PolarClean or PolarClean–acetone systems.

Combining the previously described results suggests that the finger-like membrane structure is likely due to the rapid infiltration of water in the cast film from the drop in viscosity and poorer solubility of the poly(butadiene) domain, limiting the polymer from forming a significant degree of entanglement. As PolarClean is a good solvent for both polystyrene and polyacrylonitrile, it can delay the solidification of the polymer to yield finger-like structures as well. The addition of acetone and ethanol caused some top surface densification prior to the solidification due to their higher volatility, which decreased the surface pore size. Acetone-containing membranes most likely proceed via a nucleation and ripening process. Lastly, ethanol-containing solutions can still form larger finger-like structures, like in the PolarClean case, due to the small degree of polymer entanglements, but as water diffuses into the film it can trigger instantaneous demixing related to the mixture (PolarClean–ethanol–water–ABS) being below the UCST and proceed via spinodal decomposition [44].

### 3.5. Membrane Properties and Filtration Performance

**Figure 4:**
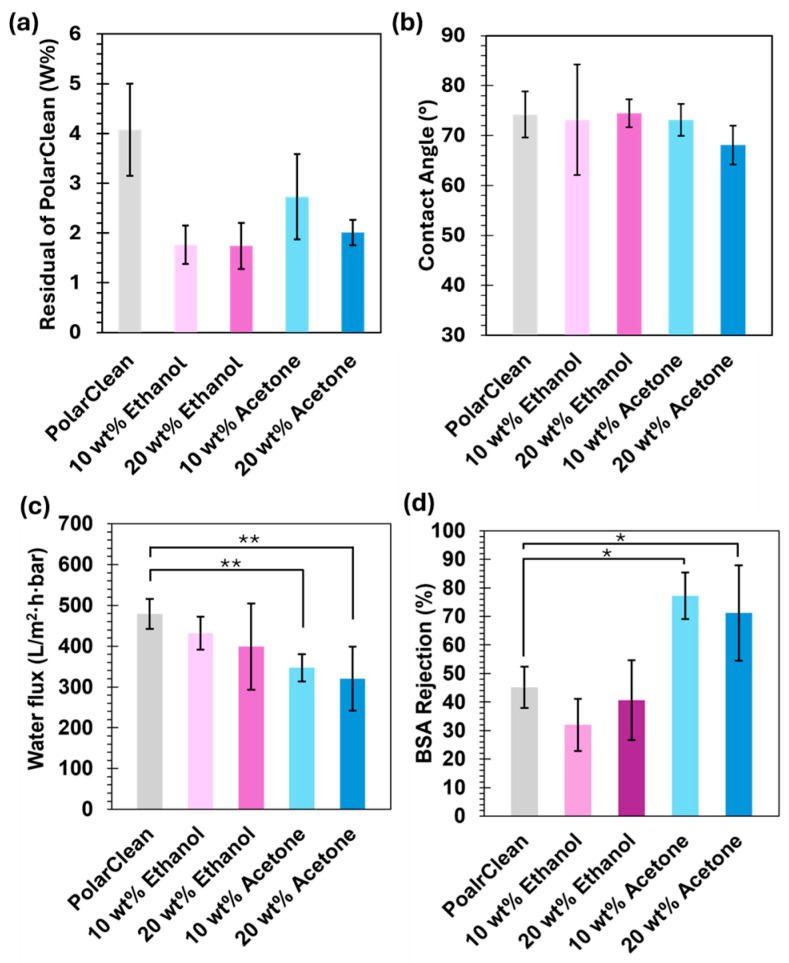
Effect of cosolvents on the (**a**) residual PolarClean in membranes, (**b**) contact angle, (**c**) water flux of membranes at 20 psi, and (**d**) corresponding BSA rejection. All data are presented as mean ± s.d. independent samples involved (**a**) *n* = 3, (**b**) *n* = 3, (**c**) *n* = 6, and (**d**) *n* = 4. Two-tailed Student’s *t*-test analyzed statistical significance in both a and b compared to the pure sample, where * *p* < 0.05, ** *p* < 0.01.

The membranes fabricated in this study were evaluated using contact angle to determine surface hydrophilicity and using NMR to determine the amount of residual PolarClean in the ABS membranes. The contact angle of the ABS membranes is shown in Figure 4. The average contact angles varied from 68 to 75 degrees, indicating a mildly hydrophilic membrane surface [7,10]. Contact angle measurements over time also show minimal changes. Another important parameter is the residual solvent in the resulting membrane. The weight percentage of residual PolarClean was calculated using ^1^H NMR, as shown in Figure 4. There was a significant decrease in residual PolarClean content after incorporation of additives. The addition of 10 and 20 wt% ethanol and acetone significantly decreased residual PolarClean from 4 wt% to around 2–2.7 wt%. We hypothesize that the drop in PolarClean residual is due to the additives reducing the solution viscosity, which allows for easier exchange of solvents during phase inversion.

Lastly, the impact of the additives on the water flux and BSA rejection was investigated, as shown in Figure 4. First, compared to ABS membranes formed by Kamelian et al., the membranes presented in this publication have approximately three times higher water flux [11]. The PolarClean-only membranes showed a low protein rejection of about 43%, likely due to the formation of larger pores on the top surface, as shown in Figure 3. The ethanol-containing meters had, similarly, a poor rejection, likely due to still relatively large pores and the presence of some defects. However, 10 and 20 wt% acetone additive concentration led to a 100 LMH bar^−1^ decrease in water flux and an increase in BSA rejection to above 70 %. This increase in rejection is likely due to acetone evaporation even under the no-evaporation time conditions, resulting in the densification of the surface layer and the formation of smaller pores, consistent with other findings and our results [51]. When compared, 20 wt% ethanol and 10 wt% acetone membranes, which were initially volatility and viscosity matched, demonstrated significant differences in membrane performance. The 20 wt% ethanol membrane showed an overall increase in variability, which could indicate some volatility but also the more complicated phase inversion process described before.

### 3.6. Impact of Evaporation Time on Membrane Formation

**Figure 5:**
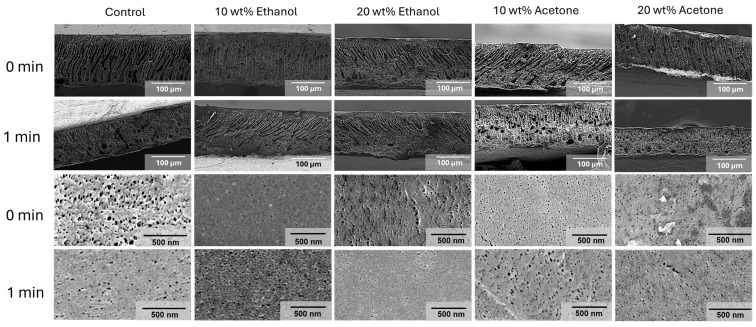
Effect of evaporation time and cosolvents on the porosity of 16 wt% ABS membranes observed with a SEM cross-section analysis.

To increase the rejection against a protein contaminant, the impact of evaporation time was studied. The SEM images in Figure 5 showed how increasing evaporation time during membrane fabrication impacted the cross-sectional structure of 16 wt% ABS membrane. In the PolarClean-only solutions, no significant impact from evaporation was expected due to the low volatility of the solvent. However, after one minute of the evaporation step, the pore structure was completely transformed from finger-like pore morphology to more of a sponge-like pore structure. The literature suggests that the sponge-like pore structure may be a result of slower demixing [41]. In our study, the delay in demixing is likely due to humidity exposure rather than the evaporation process. During the evaporation step, water vapor could adsorb into the PolarClean solutions and trigger slow phase separation. The slow water vapor-induced phase separation is supported by rapid precipitation of the polymer at a low-water content shown in the Supplementary Materials. Additionally, there is a significant decrease in membrane thickness. When looking at the solutions containing additives, we note some discrete differences. The pore structure of ethanol solutions remains relatively unchanged, with some finger-like and sponge-like domains in each. Acetone-containing solutions, on the other hand, shifted towards a more sponge-like structure after one-minute evaporation. This shift to a sponge-like structure is hypothesized to result from the densification of the top layer, leading to an overall slowdown in the solvent exchange and the formation of a sponge-like structure [41,52]. The 20% acetone containing solutions also undergo a significant decrease in thickness, supporting the significant evaporation of acetone from those solutions. The 10% and 20% ethanol in turn maintained the structure without a significant difference in the substructure potentially due to their lower volatility versus acetone.

**Figure 6:**
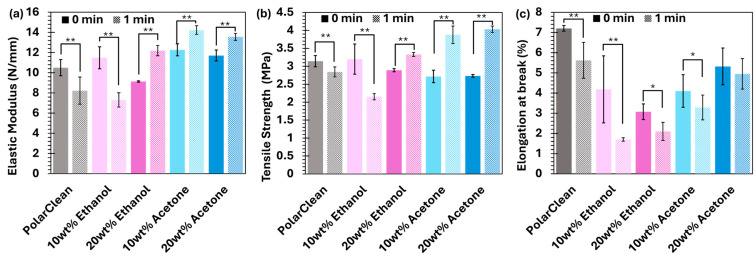
Effect of additives and evaporation time on tensile properties of 16 wt% ABS membranes with evaporation time were evaluated. The additive concentration is either 0, 10, or 20 wt%. (**a**) Elastic modulus; (**b**) tensile strength; (**c**) elongation at yield. All data are presented as mean ± s.d. n ≥ 4 independent samples. Outliers have been identified and removed via the interquartile method. Two-tailed Student’s *t*-test analyzed statistical significance in both a and b compared to the pure sample, where * *p* < 0.05, ** *p* < 0.01.

To better understand membrane morphology, the mechanical properties of the membranes were examined. Figure 6 shows the tensile strength, elastic modulus, and elongation at break for the membranes tested in this work. In the case of the PolarClean membranes, a significant decrease in all-mechanical properties are noted, which could be due to more PolarClean being left in the membrane as a result of the poorer solvent exchange and decrease in porosity. On the other hand, membranes containing 10 or 20 wt% acetone or 20 wt% ethanol increased in modulus and tensile strength with longer evaporation time. This improvement may be due to the ability of acetone and, to some extent, higher ethanol content, to slow down water penetration and partially evaporate during the one minute evaporation time, which likely gave polymer chains more time to organize and form a stronger network, as previously described in the literature [53,54,55]. The 10 wt% ethanol membranes, on the other hand, showed lower strength at 1 min of evaporation, which may be attributed to this membrane having the lowest volatility and, thus, the thinnest skin layer.

Elongation at break followed a similar trend to those of modulus and strength. PolarClean only had the highest elongation, potentially due to the plasticization effect of the residual solvent. The additive-containing membranes follow a common trend in the literature that sponge-like structures had a higher elongation at break versus those having more finger-like morphology retained by the ethanol additive membranes.

A comparison of the mechanical properties to other commonly used membrane materials is summarized in Table S3. The ABS membranes developed in this study reached tensile strengths comparable to those of polysulfone and polyether sulfone membranes. This highlights the potential of ABS as a viable material for ultrafiltration applications that require both moderate strength and flexibility.

**Figure 7:**
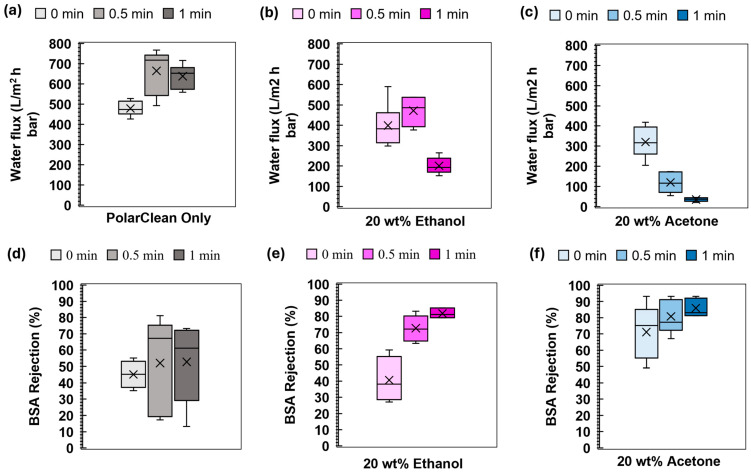
Effects of additives and evaporation time on water flux and bovine serum albumin (BSA) rejection of 16 wt% ABS membranes with evaporation time were evaluated. The cosolvents concentration of 0 and 20 wt% was plotted. (**a**) Water flux of control ABS with PolarClean without cosolvent; (**b**) water flux of ABS with PolarClean with 20 wt% Ethanol cosolvent; (**c**) water flux of ABS with PolarClean with 20 wt% Acetone as cosolvent; (**d**) BSA rejection of control ABS with PolarClean without cosolvent; (**e**) BSA rejection of ABS with PolarClean with 20 wt% Ethanol cosolvent; (**f**) BSA rejection of ABS with PolarClean with 20 wt% acetone cosolvent. All data are presented as mean ± s.d., n = 4 independent samples. Average is reported via x on the box, and the median is reported via a line in the box. Outliers were identified and removed via the interquartile method.

Lastly, we evaluated the impact of evaporation on the filtration performance of membranes for application in potential protein purification. Figure 7 shows that the PolarClean only system increased in the BSA rejection and in variability of the performance, which could be attributed to the local variations in the phase inversion process triggered by humidity. Interestingly, an increase in permeability was also noted, despite the decrease in the porosity of the membrane. The increased water flux may be due to a drop in membrane thickness, as noted in the Supplementary Materials. Increasing evaporation time for ethanol additives resulted in an increase in BSA rejection with a loss in permeability, albeit not as significant as those with acetone. Acetone-containing samples had the most significant trade-off in permeability versus rejection and showed the highest rejections. Both of these trade-offs may be a result of a dense layer formation near the top surface. For the matched volatility and viscosity solutions, 20% ethanol and 10% acetone, the permeability was not matched, indicating that the ethanol system was able to maintain a higher permeability due to differences in the phase inversion process.

## 4. Conclusions

ABS membranes were successfully fabricated using a green solvent, PolarClean, and water as the non-solvent bath. The membrane performance was carefully tuned using ethanol and acetone additives. The additives impacted the surface pore size and pore architecture throughout the membrane. It was found that ethanol-containing solutions had a UCST when a small amount of water was added, which supported the spinodal decomposition pathways for these membranes, while acetone membranes may have proceeded through a nucleation and growth pathway primarily. Protein rejection was increased by increasing evaporation time, which led to the densification of the top surface and initiated a humidity-mediated phase inversion process in the case of PolarClean-only membranes. Additionally, all membranes’ structure and performance changed significantly which in turn increased BSA rejection and decreased water flux. In brief, it was concluded that adding additives to a green solvent, such as PolarClean, could alter the precipitation path of the membrane and its susceptibility to humidity.

## Figures and Tables

**Table 1:** CHEM 21 selection guide ranking of solvents involved in the study.

	BP (°C)	FP (°C)	Viscosity (mPa·s) ^a^	Vapor Pressure (Pa) ^b^	Worst H3xx ^c^	H4xx	Safety Score	Health Score	Environmental Score	Ranking
PolarClean ^d^	280	146	9.82	1	H319	None	2	2	1	Recommend
Ethanol ^e^	78	13	1.1	8200	H319	None	4	3	3	Recommend
Acetone ^e^	56	−18	0.32	30,800	H319	None	5	3	3	Recommend

BP, boiling point; FP, flash point. The scores have the following ranking: scores 1–3 are recommended, scores 4–6 are problematic, and scores above 7 are hazardous. ^a^ Measurement at 20 °C; ^b^ measurement at 25 °C; ^c^ only the highest scoring statements are shown. Clear definitions can be seen in Prat et al. [29]; ^d^ BP, FP, viscosity, and vapor pressure values provided by the manufacturer [37]; ^e^ values from Prat et al. and NIST [29].

**Table 2:** Properties of the solutions used in the study.

ABS wt/wt%	Solvent Composition	RED ^a^	Viscosity ^b^ (mPa·s)	Initial Volatility ^c^ (%)
16	PolarClean	0.89	1013 ± 3	0
16	10 wt% Ethanol 90 wt% PolarClean	0.90	551 ± 20	1 ± 0.8
16	20 wt% Ethanol 80 wt% PolarClean	0.93	467 ± 33	2 ± 1.6
16	10 wt% Acetone 90 wt% PolarClean	0.89	487 ± 52	2 ± 0.8
16	20 wt% Acetone 80 wt% PolarClean	0.90	384 ± 27	5 ± 0.5

^a^ Calculated with parameters provided in the Supplementary Materials; ^b^ measurement at 25 °C; ^c^ measurement at 20 °C.

## Data Availability

The original data presented in the study are openly available in Open Science Framework Repository at https://osf.io/r2wc6/?view_only=e282fc577da445d5bb225b9b0d061295 (accessed on 1 May 2025).

## Supplementary Materials

The following supporting information can be downloaded at: https://www.mdpi.com/article/10.3390/membranes15060181/s1. Scheme S1. Fabrication process of ABS membrane using nonsolvent-induced phase separation (NIPS) technique Created in BioRender. Figure S1: NMR spectra of ABS membrane made of PolarClean without diluents; Figure S2: NMR spectra of ABS membrane made of PolarClean with 10 wt% Ethanol.; Figure S3: NMR spectra of ABS membrane made of PolarClean with 20 wt% Ethanol.; Figure S4: NMR spectra of ABS membrane made of PolarClean with 10 wt% Acetone.; Figure S5: NMR spectra of ABS membrane made of PolarClean with 20 wt% Acetone.; Table S1: Solvent Properties and RED scores. When RED <1 the solvent is identified as a good solvent; Table S2: (a) Hansen solubility parameter distances of copolymer ABS, homopolymer polystyrene (PS) polybutadiene (PB) and Polyacrylonitrile (PAN) (PS,PB,PAN)), with pure PolarClean, ethanol, acetone, and with blending of PolarClean with 10,20 wt% of acetone or ethanol (b) Hansen solubility parameter distances of copolymer ABS, with pure PolarClean (control) and with blending of PolarClean with 10, 20 wt% of acetone or ethanol before and after adding 10% water. R0 is radius of interaction for the ABS = 9.4, PS = 10.3, PB = 6.55, PAN = 11.2 Mpa^0.5^; Figure S6: ABS 3D solubility comparison a) before adding 10% of water b) after adding 10% water, this figure highlights how the system contains ethanol became out of the radius and become immiscible after addition the water compare with other system.; Figure S7: Viscosity curves vs. shear rate of 16 wt% ABS in PolarClean (blue line) and of 16 wt% ABS in PolarClean with 20 wt% of acetone (blue line) or 20% ethanol (magenta line) with the addition of 5 wt% water prior to rheology analysis.; Figure S8: 16 wt% Polystyrene in PolarClean (a) before, (b) immediately after the addition of 2 drops of water (~60 mg), and (c) after 24 h; 16 wt% ABS in PolarClean (d) before, (e) immediately after addition of 5 wt% water, (f) after 24 h.; Figure S9: 16wt% Polystyrene in PolarClean with 20 wt% ethanol (a) before, (b) immediately after addition of 2 drops of water (~60 mg), and (c) after 24 h; 16 wt% ABS in PolarClean + 20 wt% ethanol (d) before, (e) immediately after addition of 5 wt% water and (f) after 24 h; Figure S10: 16 wt% ABS in PolarClean with 20 wt% Acetone (a) before; (b) after addition of 5 wt% water; and (c) after 24 h and added with 2 drops (~60 mg) of water.; Figure S11: The 16 wt% of ABS solution with 20 wt% ethanol after 24 h of addition of 5 wt% water at room temperature vs 50 °C; Here, the ABS ethanol PolarClean solutions containing 5 wt% of water had a thermal transition at 50 °C from a soluble to an insoluble mixture. This temperature-based response suggests the presence of upper boundary solution temperature or upper critical solution temperature behavior confirming the viscosity results. Interestingly, in different polymer-solvent combinations used in this study, the same gelation/solidification behavior was not displayed as observed in the ABS, PolarClean, ethanol, and water systems. UCST transitions have long been correlated with intermolecular interactions triggering phase separation. At these low volume fractions of water, water molecules exist as free molecules, competing for hydrogen bonding with PolarClean, and ethanol.; Figure S12: The volatility of pure PolarClean, Ethanol, Acetone, and blend solvent; Figure S13: Differential scanning calorimetry of pure poly(acrylonitrile-butadiene-styrene) heated to 150 °C and cooled to −90 °C at a rate of 10 °C/min for 2 cycles to determine glass transition temperature via half height analysis. The curve shown displays the second heating ramp.; Figure S14: GPC 1 mL/min ethyl acetate: Conventional calibration to Polystyrene. Mn = 80050 g/mol ± 17.8%. PDI = 1.645 ± 25.2%; Figure S15: Light microscopy images of solutions, 2h after synthesis; Figure S16: FTIR-ATR of 16wt% ABS membranes with pure PolarClean or diluents (10/20 wt% ethanol, 10/20wt% acetone) (a) the full spectra (b) zoomed in spectra on the region of a unique peak corresponding to PolarClean residual in the membrane indicating that all membranes have some PolarClean left over in the structure; Figure S17: Contact angle of ABS membranes measured using a sessile drop analysis. (a) Contact angle of membranes with zero minutes of solvent evaporation time. (b) Contact angle of membranes with 0.5 min of solvent evaporation time. (c) Contact angle of membranes with 1 min of solvent evaporation time. The contact angle of all membranes was below 90°, signifying good wettability and thus, hydrophilic nature of the membrane surface. 20% Acetone with evaporation times of 0 and 1 min had the lowest contact angle. However, all membranes had contact angles comparable or below that of the control membranes.; Figure S18: ABS 1H NMR spectra showing a composition of 48% poly(styrene), 14% poly(butadiene), 38% poly(acrylonitrile) performed in CDCl3.; Figure S19: Average pore size of the membrane. SEM images of top surfaces, previously coated with 3 nm of Pt to increase conductivity, were analyzed via MATLAB and ImageJ to detect and quantity number of pores. The pores less than 5 nm in diameter were excluded.; Figure S20: The impact of the 1 min evaporation time on the ABS membrane porosity. The porosity dropped for all membranes except for ethanol containing solutions which undergo the spinodal decomposition.; Figure S21: The impact of the 1 min evaporation time on the ABS membrane thickness; membrane thickness decreased after one minute of evaporation, with the greatest drop in 20 wt% acetone due to solvent volatility and early surface solidification. In the PolarClean-only membrane, humidity likely triggered early phase separation, limiting swelling during immersion and resulting in a thinner structure.; Table S3. Comparative Mechanical Properties of Common Flat Membrane Polymers from the Literature; Figure S22: The top surface and cross section SEMs of the cast membranes with zero evaporation time; Figure S23: The top surface and cross section SEMs of the cast membranes with one min evaporation time.

## Abbreviations

The following abbreviations are used in this manuscript:

ABS	Poly(acrylonitrile-butadiene-styrene)
NIPS	Non-solvent-induced phase separation
