# Supplementary material for: Impact of Additives on Poly(acrylonitrile-butadiene-styrene) Membrane Formation Process Using Non-Solvent-Induced Phase Separation
Source: Membranes (Basel). 2025 Jun 16;15(6):181. doi: 10.3390/membranes15060181 (PMC12195195; doi:10.3390/membranes15060181)
Supplement: Supplementary file 1 [file membranes-15-00181-s001.zip › membranes-3648686-supplementary.pdf]

Methods:

Fourier transform infrared spectroscopy

A Thermo Scientific Nicolet iS50 Attenuated Total Reflection-Fourier Transform Infrared (ATR-FTIR) spectrometer was used to analyze the membranes' surface chemical structure. Before analysis, each membrane sample was dried overnight. Background signal was collected before sample analysis. Each spectrum presented resulted from 32 accumulations obtained with a resolution of 4 cm−1 with air as the background.

Residual PolarClean detection on the ABS membrane structure by NMR

To analyze the chemical structure of the synthesized ABS membrane and detect any residual PolarClean, NMR spectra were measured using a Bruker NMR spectrometer. Each sample was weighed and dissolved in 700 ul of deuterated chloroform. Samples were spiked with 50 µL of dichloromethane (DCM) to detect the residual solvent. Proton (1H) NMR spectra were obtained with 32 scans to ensure a sufficient signal-to-noise ratio. Distinct peaks corresponding to ABS and PolarClean were identified, ensuring that selected peaks do not overlap with others. For PolarClean, the methyl peak was located and integrated to quantify the residual amount of solvent. This process was repeated for all samples to determine the residual PolarClean content.

Calculations:

$$Molar\ ratio = \frac{\frac{DCM\ integration}{\#\ H\ on\ DCM}}{\frac{PolarClean\ integration}{\#\ H\ on\ PolarClean}} = \frac{\frac{2}{2}}{\frac{PolarClean\ integration}{6}}$$

$$= \frac{6}{PolarClean\ integration}$$

$$moles\ Polar\ Clean = \frac{moles\ DCM * PolarClean\ integration}{6}$$

$$wt\% = \frac{moles\ PolarClean * MW\ PolarClean}{sample\ mass} * 100\%$$

Viscosity measurement using Rheometer

The viscosity of the ABS in PolarClean solution after different addition of acetone and ethanol was measured using a Rheometer from TA Instrument. Three vials of 16 wt% ABS dissolved in pure PolarClean were prepared. One vial was then added with 20 wt% acetone, and another was added with 20 wt% ethanol. The solutions were mixed at 40 C and 250 RPM overnight until thoroughly homogeneous, then let to cool completely at room temperature for 24 hours while continue stirring. Fifty µL of DI water was added to each vial and swirled around five minutes before putting it on the rheometer plate. Shear rate ranging from 0 to 80 s^{-1} was applied.

	ABS (g)	Polarclean (g)	Acetone (g)	EtOH (g)	Total weight (g)
Vial 1	0.16	0.84	-	-	1.00

Vial 2	0.16	0.64	0.20	-	1.00
Vial 3	0.16	0.64	-	0.20	1.00

Light microscopy for imaging

Nine samples were prepared with the following compositions. Three vials were PolarClean added with 20 wt% ethanol (vial 4, 5, 6) and another three were PolarClean added with 20 wt% acetone (vial 7, 8, 9), while another three only contain PolarClean (vial 1, 2, 3). Vials 2, 3, 5, 6, 8, and 9 were added with 16 wt% ABS in 40 C and 250 RPM and were left to cool in room temperature for 24 hours while continue being stirred. Three of these vials (vial 2, 5, 8) as well as vials 1, 4, 7 were then added with 100 uL of water and were left for 2 hours prior to microscope imaging. The image was recorded at 10x and 20x magnification.

PolarClean + 100 uL Water	PolarClean + 20 wt% Ethanol + 100 uL Water	PolarClean + 20 wt% Acetone + 100 uL Water
PolarClean + 100 uL Water + ABS	PolarClean + 20 wt% Ethanol + 100 uL Water + ABS	PolarClean + 20 wt% Acetone + 100 uL Water + ABS
PolarClean + ABS	PolarClean + 20 wt% Ethanol + ABS	PolarClean + 20 wt% Acetone + ABS

Volatility of dope solution

To investigate the volatility of a casting solution, we placed 3 mL of PolarClean, a diluent, or a blend of PolarClean with 10-20 wt% of another diluent onto a glass petri dish. We measured the weight change over time using an analytical balance (Scout™ Pro 200g, Ohaus, USA). The experiment was conducted for ten minutes at room temperature in a fume hood with moderate airflow, simulating the conditions during membrane casting. Volatility was calculated based on the change in weight over time.

Contact Angle Measurements

To determine the wettability of the membrane surfaces, a contact angle goniometer (Krüss Drop Shape Analyzer) via the sessile drop method, was used. Six, approximately 1.5″ x 2″ rectangles, were cut from various sections of the dried membranes and adhered to a glass slide using double sided tape. The slide was placed on the stage and the stage was manually aligned until the bottom of the membrane was in-line with the baseline for the integrated camera, observed using the Krüss desktop application. A 2 μL deionized water (UltraPure Type II) was manually dispensed onto the membrane surface, and the contact angle was automatically recorded using an integrated camera and image analysis software (need). A Sessile Drop analysis using a Krüss Drop Shape Analyzer was used to automatically measure the contact angle, recorded at a rate of two frames per second for 40 seconds. The contact angle was automatically calculated using the built0in image software analysis. All measurements were taken at room temperature with ambient humidity. For each sample, six individual measurements were taken and the resulting contact angles from each were averaged and the standard deviations were calculated using Microsoft Excel.

Image analysis

To determine the pore size of the top surface pores, image analysis using MATLAB and ImageJ. MATLAB script is published and validated versus manual measurements.[1] N=5 was used for images. Acetone 20% was not successfully analyzed by the pore script and was instead manually analyzed with ImageJ.

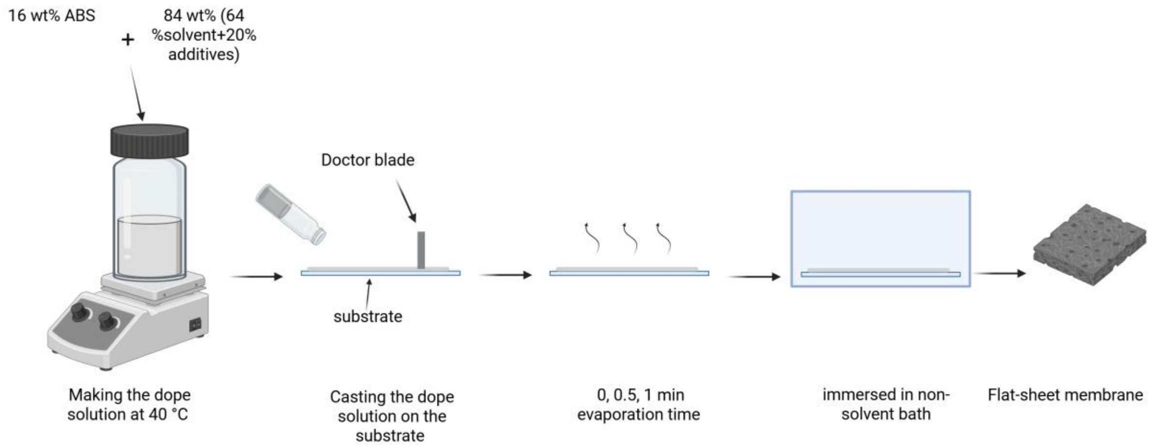

Scheme 1. Fabrication process of ABS membrane using nonsolvent-induced phase separation (NIPS) technique Created in BioRender.

Results:

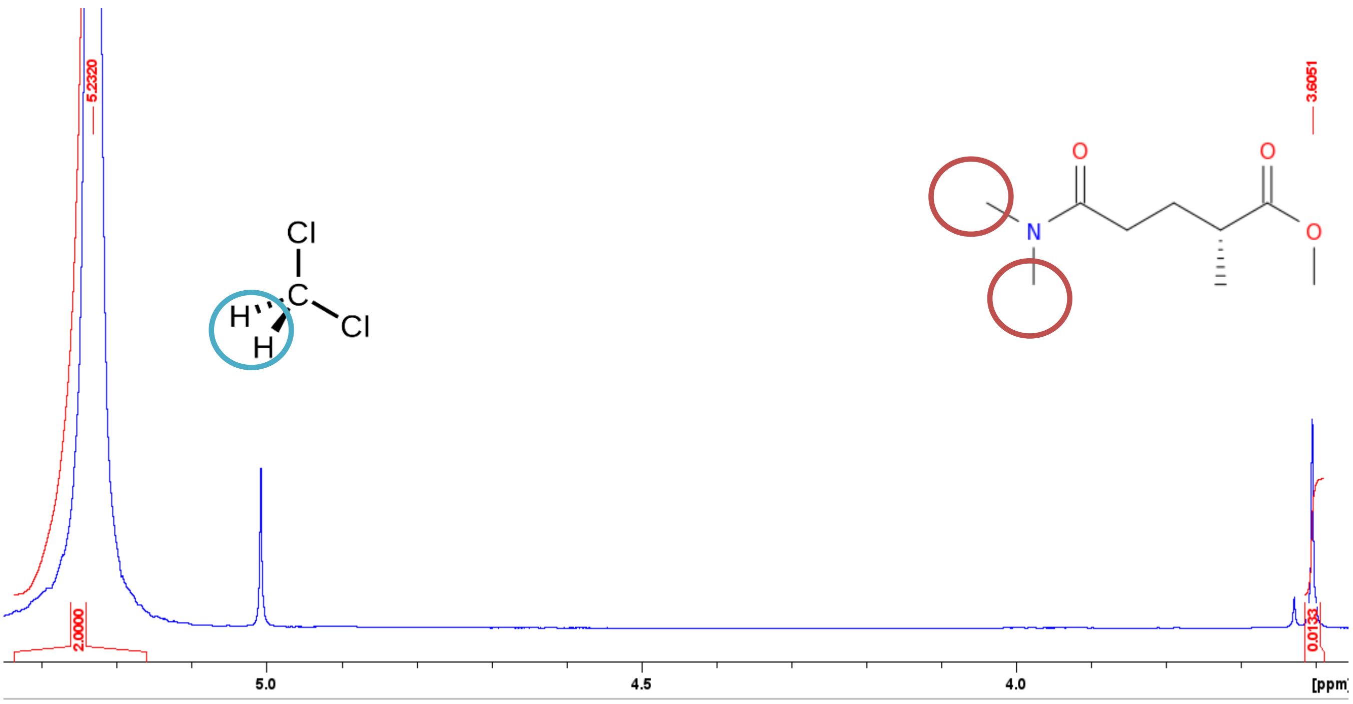

Figure S1. NMR spectra of ABS membrane made of PolarClean without diluents.

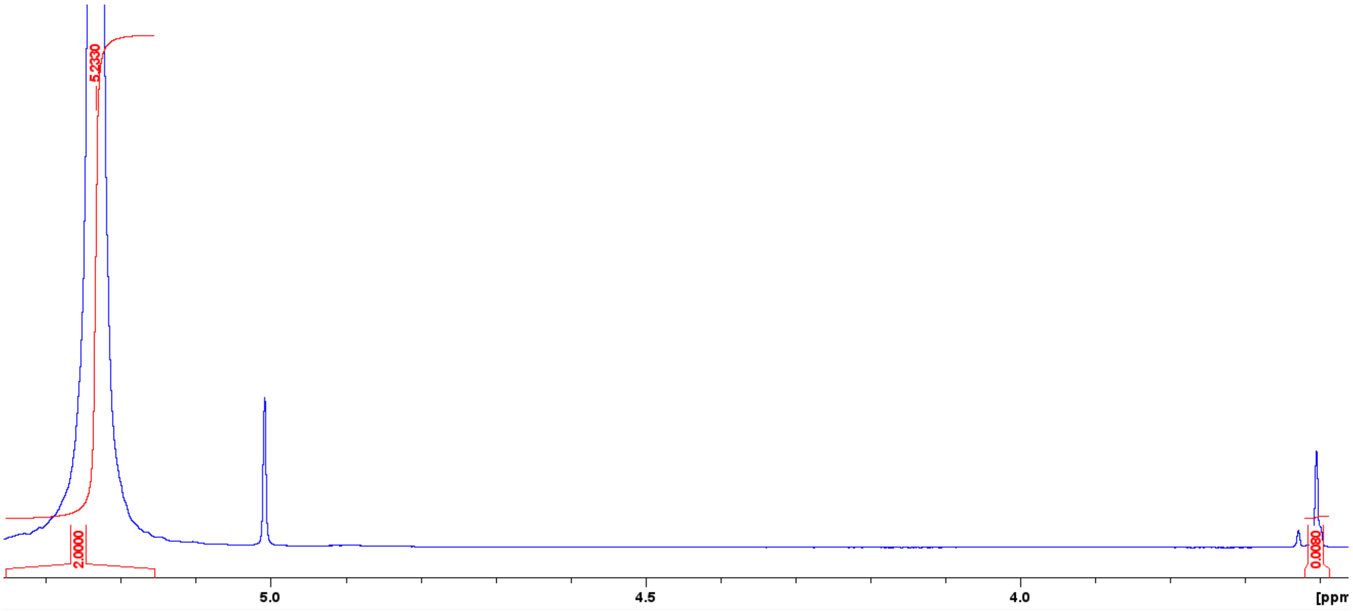

Figure S2. NMR spectra of ABS membrane made of PolarClean with 10 wt% Ethanol.

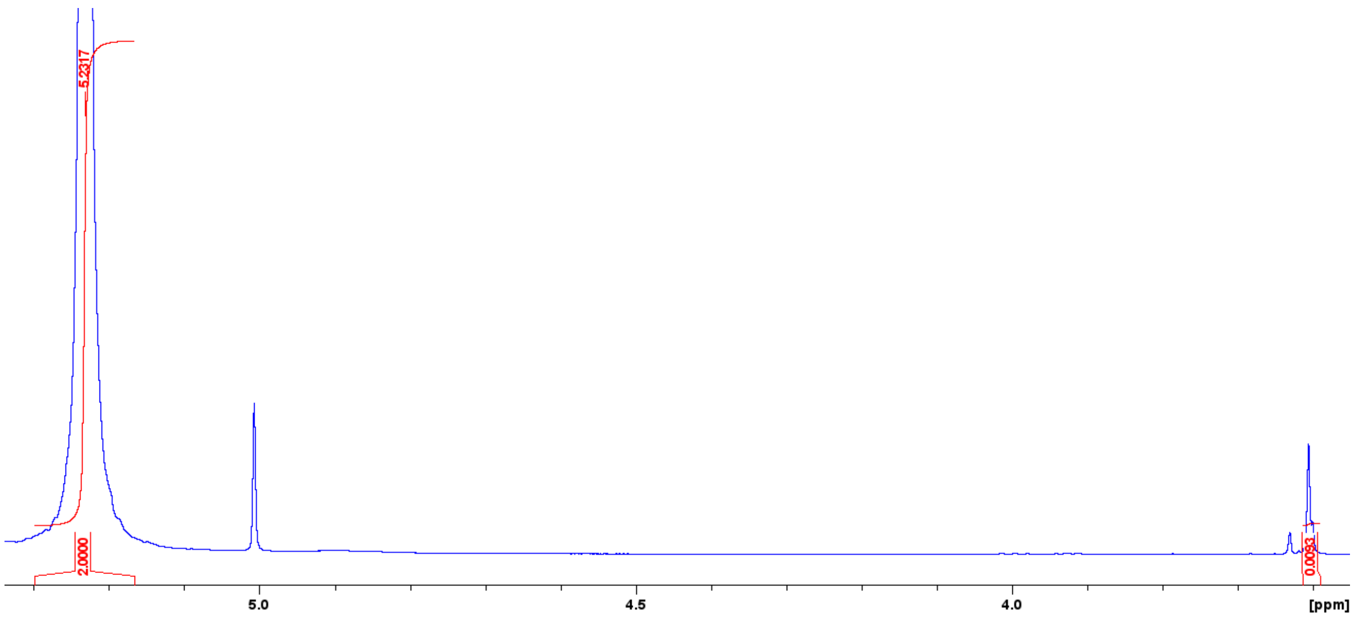

Figure S3. NMR spectra of ABS membrane made of PolarClean with 20 wt% Ethanol.

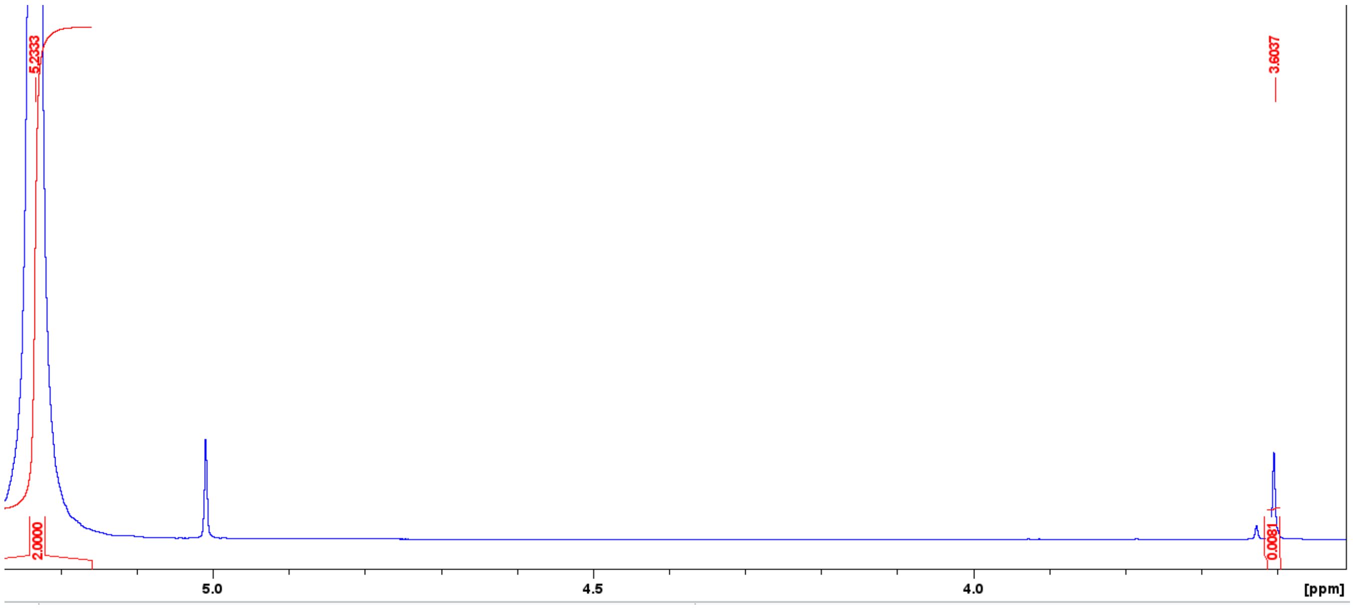

Figure S4. NMR spectra of ABS membrane made of PolarClean with 10 wt% Acetone.

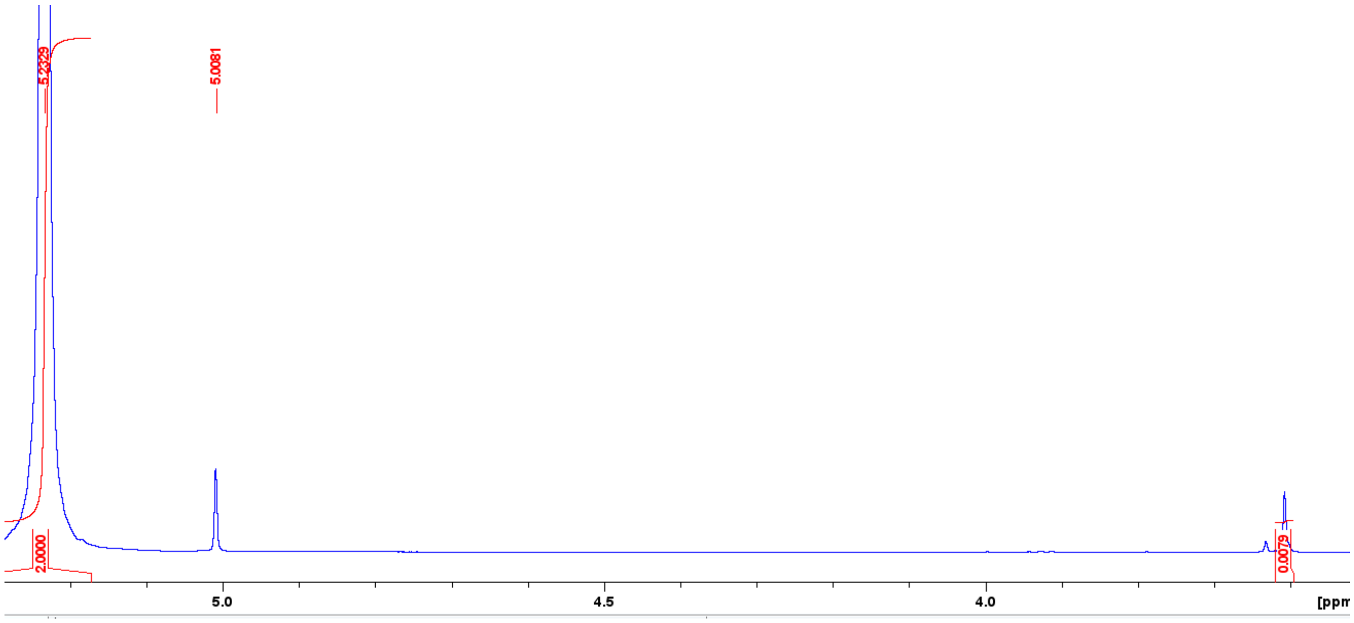

Figure S5. NMR spectra of ABS membrane made of PolarClean with 20 wt% Acetone.

Table S1. Solvent Properties and RED scores. When RED <1 the solvent is identified as a good solvent.

	**PolarClean**	**γ-Valerolactone**	**Cyrene**
Vapor pressure (pa)	1	37	14.4
Viscosity (mPa S)	9.82	0.89	14.5
RED	0.89	1.59	1.17

Table S2: a) Hansen solubility parameter distances of copolymer ABS, homopolymer polystyrene (PS) polybutadiene (PB) and Polyacrylonitrile (PAN) (PS,PB,PAN)), with pure PolarClean, ethanol, acetone, and with blending of PolarClean with 10,20 w% of acetone or ethanol b) Hansen solubility parameter distances of copolymer ABS, with pure PolarClean (control) and with blending of PolarClean with 10,20 w% of acetone or ethanol before and after adding 10% water.R0 is radius of interaction for the ABS= 9.4 , PS=10.3, PB= 6.55, PAN=11.2 $Mpa^{0.5}$[2-4]

(a)

		Polar clean	Acetone	ETOH	10 wt% ETOH	20 wt% ETOH	10% Acetone	20% Acetone
Vapor pressure (pa)		1	24000	5950				
Viscosity (mPa S)		9.82	0.32	1.1				
				Solubility parameter				
	ABS							
δd(Mpa^1/2)	16.3	15.8	15.5	15.8	15.8	15.8	15.77	15.74
δp(Mpa^1/2)	2.7	10.7	10.4	8.8	10.51	10.32	10.67	10.64
δh(Mpa^1/2)	7.1	9.2	7	19.4	10.22	11.24	8.98	8.76
δT(Mpa^1/2)	17.98	21.18	19.94	26.52	21.55	21.97	21.05	20.92
Ra(Mpa^1/2)		8.33	7.87	13.77	8.47	8.73	8.26	8.19
RED		0.89	0.84	1.46	0.90	0.93	0.88	0.87
	PS							
δd(Mpa^1/2)	20.31	15.8	15.5	15.8	15.8	15.8	15.77	15.74
δp(Mpa^1/2)	8.95	10.7	10.4	8.8	10.51	10.32	10.67	10.64
δh(Mpa^1/2)	3.15	9.2	7	19.4	10.22	11.24	8.98	8.76
δT(Mpa^1/2)	22.42	21.18	19.94	26.52	21.55	21.97	21.05	20.92
Ra(Mpa^1/2)		11.00	10.46	18.59	11.57	12.19	10.93	10.86
RED		1.07	1.02	1.80	1.12	1.18	1.06	1.05
	PB							
δd(Mpa^1/2)	17.53	15.8	15.5	15.8	15.8	15.8	15.77	15.74
δp(Mpa^1/2)	2.25	10.7	10.4	8.8	10.51	10.32	10.67	10.64
δh(Mpa^1/2)	3.42	9.2	7	19.4	10.22	11.24	8.98	8.76
δT(Mpa^1/2)	18.00	21.18	19.94	26.52	21.55	21.97	21.05	20.92
Ra(Mpa^1/2)		10.81	9.78	17.61	11.24	11.76	10.69	10.57
RED		1.65	1.49	2.69	1.72	1.80	1.63	1.61
	PAN							
δd(Mpa^1/2)	19.44	15.8	15.5	15.8	15.8	15.8	15.77	15.74
δp(Mpa^1/2)	10.48	10.7	10.4	8.8	10.51	10.32	10.67	10.64
δh(Mpa^1/2)	5.27	9.2	7	19.4	10.22	11.24	8.98	8.76
δT(Mpa^1/2)	22.70	21.18	19.94	26.52	21.55	21.97	21.05	20.92
Ra(Mpa^1/2)		8.28	8.07	15.98	8.80	9.42	8.23	8.18
RED		0.74	0.72	1.43	0.79	0.84	0.73	0.73

(b)

		Before adding 10% of the water				
	ABS	Control	10 % ETOH	20 % ETOH	10% Acetone	20% Acetone
δd(Mpa^1/2)	16.30	15.80	15.80	15.80	15.83	15.86
δp(Mpa^1/2)	2.70	10.70	10.51	10.32	10.76	10.82
δh(Mpa^1/2)	7.10	9.20	10.22	11.24	9.18	9.16
δT(Mpa^1/2)	17.98	21.18	21.55	21.97	21.23	21.28
Ra(Mpa^1/2)		8.33	8.47	8.73	8.38	8.42
RED		0.89	0.90	0.93	0.89	0.90

		After adding 10% of the water				
	ABS	Control	10 % ETOH	20 % ETOH	10% Acetone	20% Acetone
δd(Mpa^1/2)	16.30	15.77	15.77	15.77	15.79	15.82
δp(Mpa^1/2)	2.70	11.23	11.14	10.95	11.26	11.32
δh(Mpa^1/2)	7.10	12.51	13.02	14.04	12.50	12.48
δT(Mpa^1/2)	17.98	23.05	23.29	23.78	23.07	23.11
Ra(Mpa^1/2)		10.16	10.36	10.83	10.17	10.21
RED		1.08	1.10	1.15	1.08	1.09

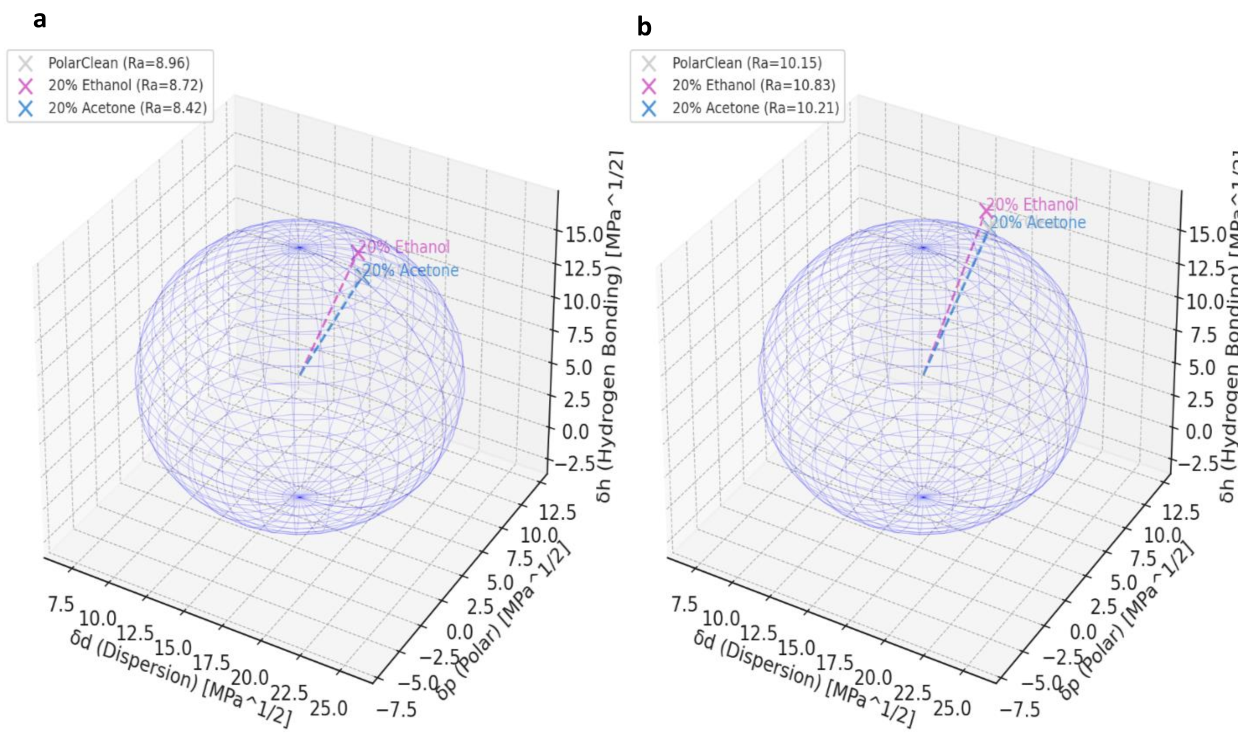

Figure S6: ABS 3D solubility comparison a) before adding 10% of water b) after adding 10% water, this figure highlights how the system contains ethanol became out of the radius and become immiscible after addition the water compare with other system.

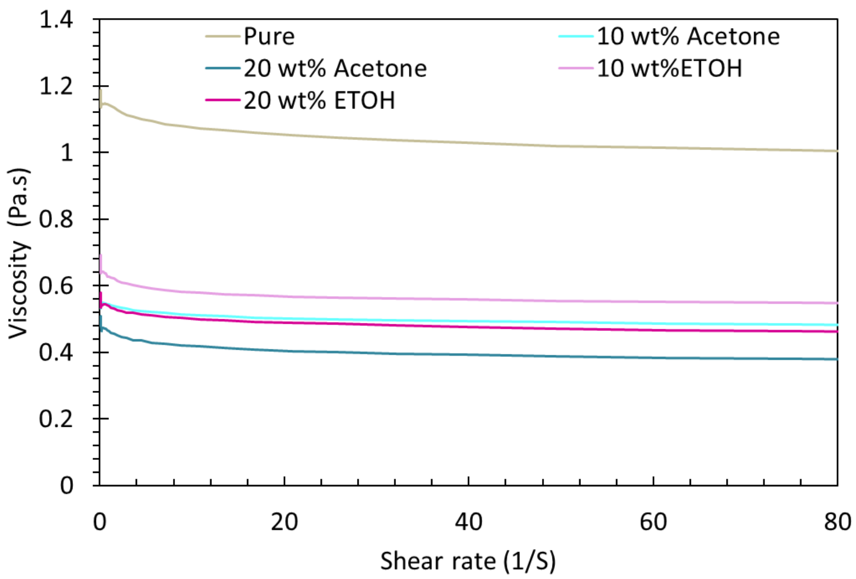

Figure S7: Viscosity curves vs. shear rate of 16 wt% ABS in PolarClean (blue line) and of 16 wt% ABS in PolarClean with 20 wt% of acetone (blue line) or 20% ethanol (magenta line) with the addition of 5 wt% water prior to rheology analysis.

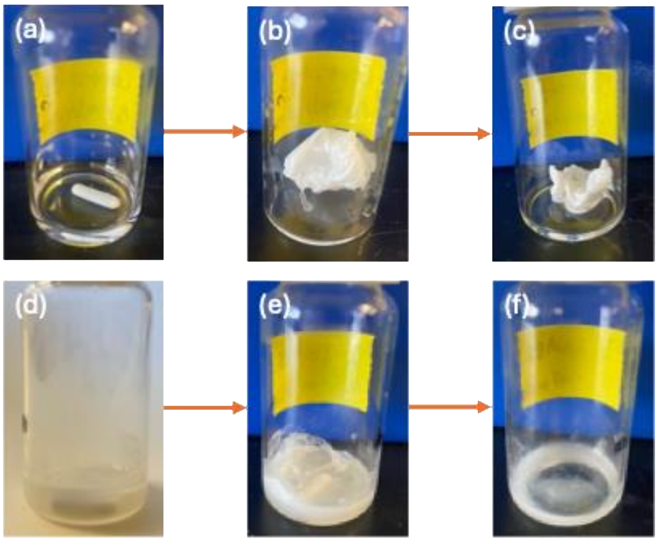

Figure S8. 16 wt% Polystyrene in PolarClean (a) before, (b) immediately after the addition of 2 drops of water (~60 mg), and (c) after 24 hours; 16 wt% ABS in PolarClean (d) before, (e) immediately after addition of 5 wt% water, (f) after 24 hours.

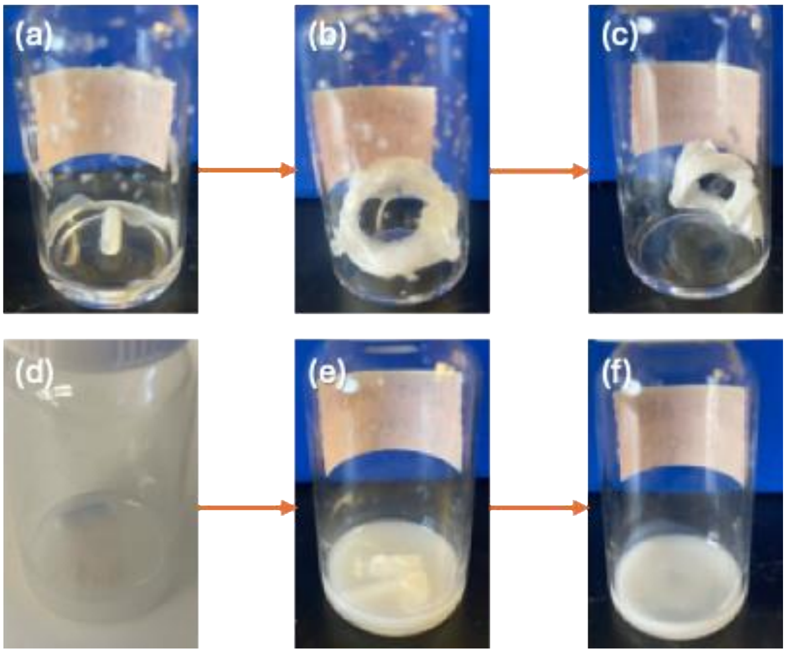

Figure S9. 16wt% Polystyrene in PolarClean with 20 wt% ethanol (a) before, (b) immediately after addition of 2 drops of water (~60 mg), and (c) after 24 hours; 16 wt% ABS in PolarClean + 20 wt% ethanol (d) before, (e) immediately after addition of 5 wt% water and (f) after 24 hours.

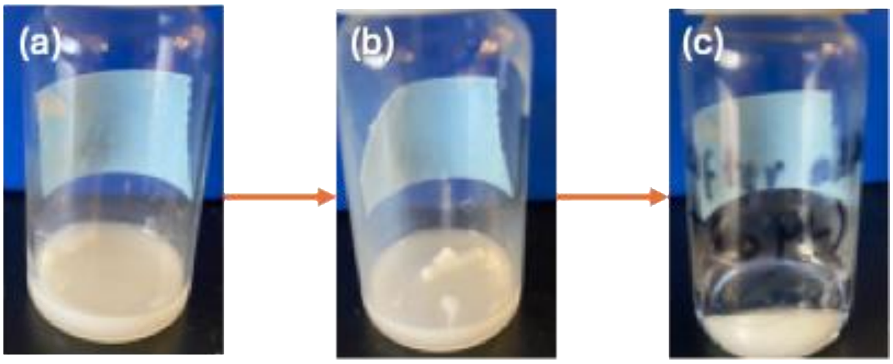

Figure S10. 16 wt% ABS in Polarclean with 20 wt% Acetone (a) before; (b) after addition of 5 wt% water; and (c) after 24 hours and added with 2 drops (~60 mg) of water.

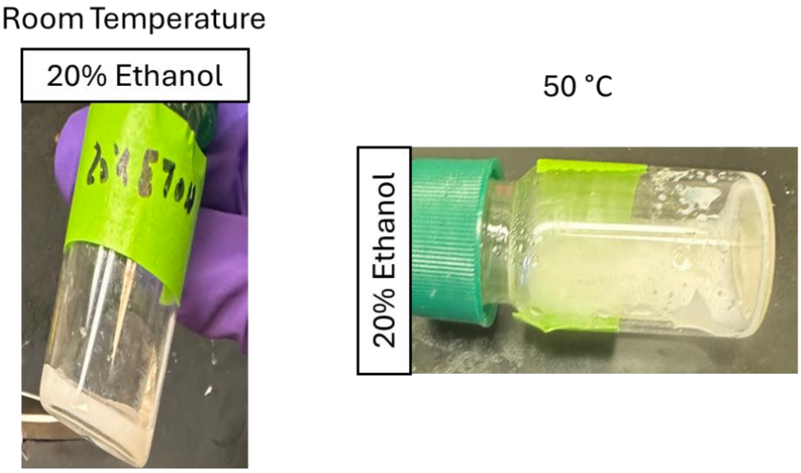

Figure S11. The 16 wt% of ABS solution with 20 wt% ethanol after 24 hours of addition of 5 wt% water at room temperature vs 50 °C; Here, the ABS ethanol PolarClean solutions containing 5 wt% of water had a thermal transition at 50°C from a soluble to an insoluble mixture. This temperature-based response suggests the presence of upper boundary solution temperature or upper critical solution temperature behavior confirming the viscosity results. Interestingly, in different polymer-solvent combinations used in this study, the same gelation/solidification behavior was not displayed as observed in the ABS, PolarClean, ethanol, and water systems. UBST transitions have long been correlated with intermolecular interactions triggering phase separation.[5, 6] At these low volume fractions of water, water molecules exist as free molecules, competing for hydrogen bonding with PolarClean, and ethanol.[7].

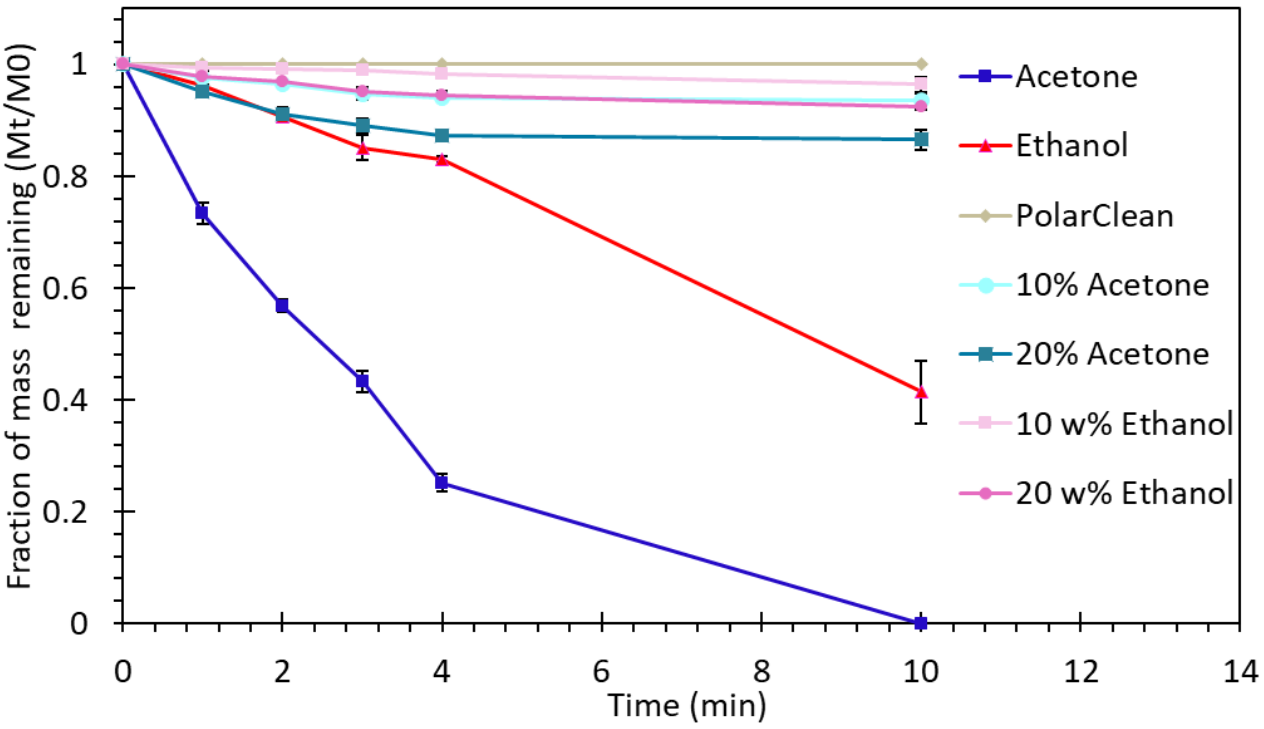

Figure S12. The volatility of pure PolarClean, Ethanol, Acetone, and blend solvents.

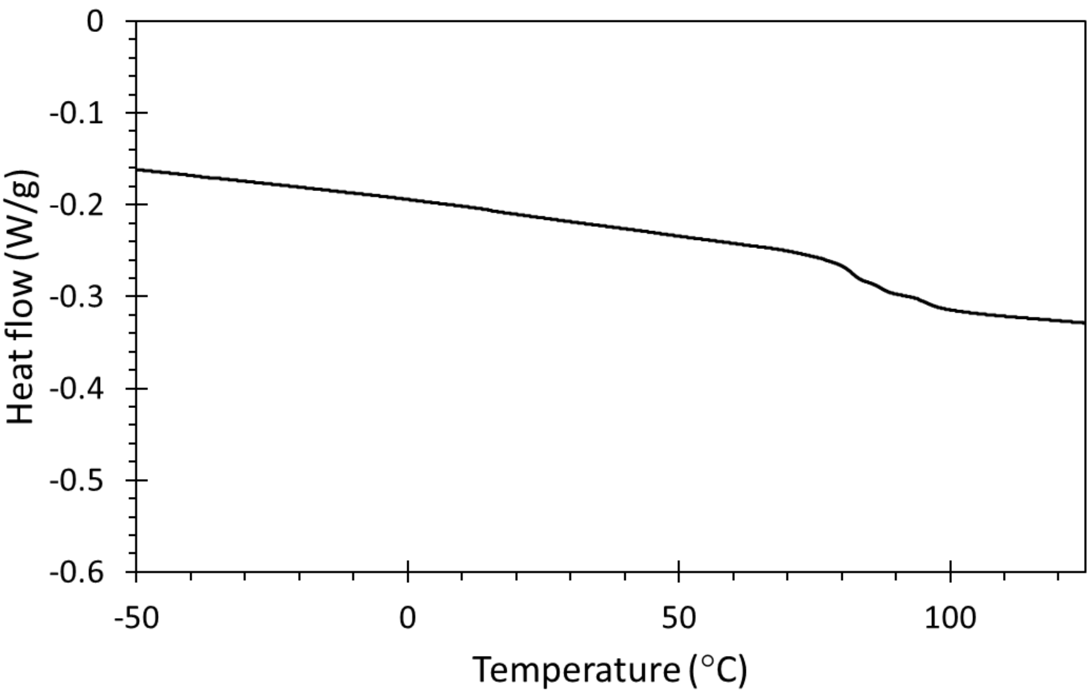

Figure S13. Differential scanning calorimetry of pure poly(acrylonitrile-butadiene-styrene) heated to 150 °C and cooled to -90 °C at a rate of 10 °C/min for 2 cycles to determine glass transition temperature via half height analysis. The curve shown displays the second heating ramp.

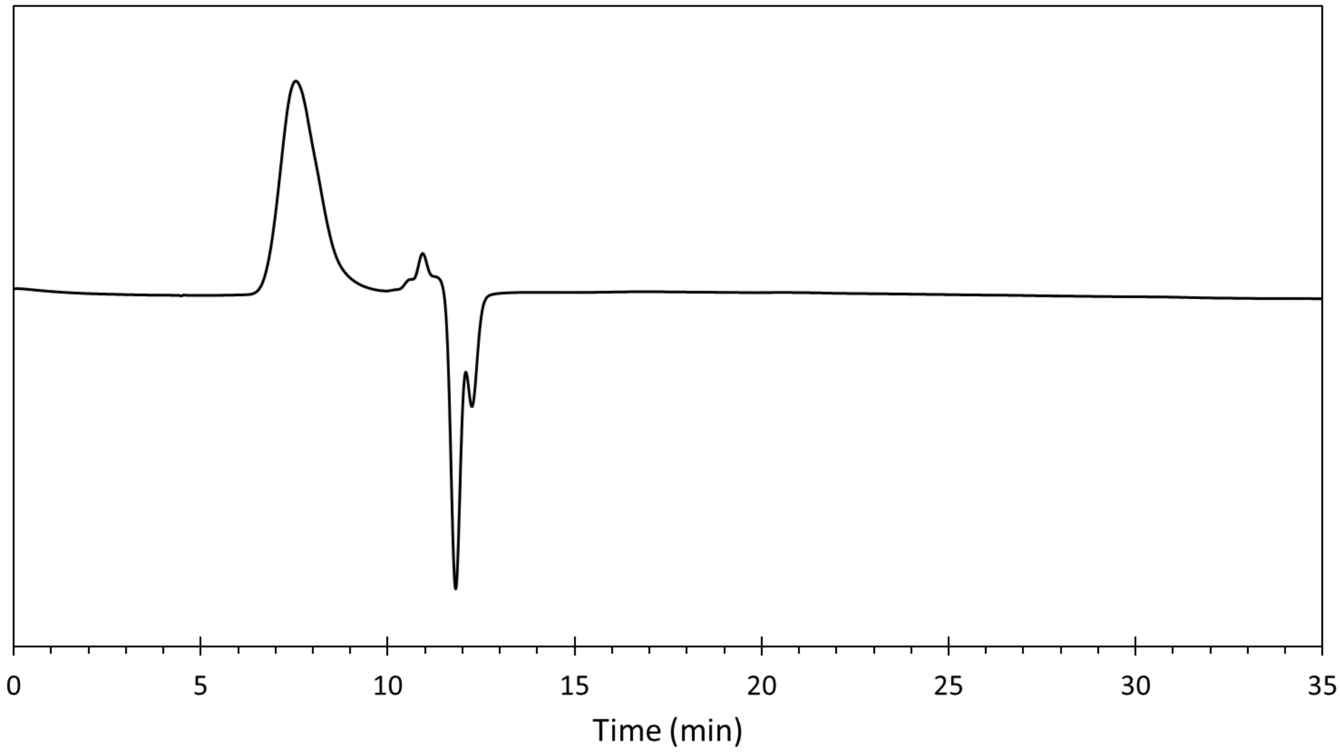

Figure S14. GPC 1 ml/min ethyl acetate: Conventional calibration to Polystyrene. M_n=80050 g/mol ±17.8%. PDI=1.645± 25.2%.

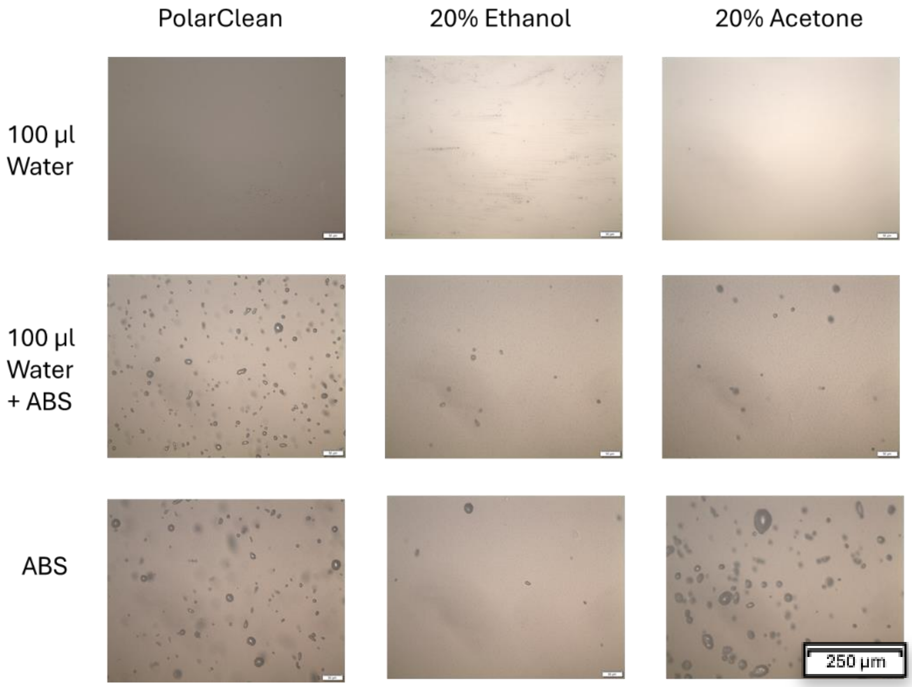

Figure S15. Light microscopy images of solutions, 2hrs after synthesis.

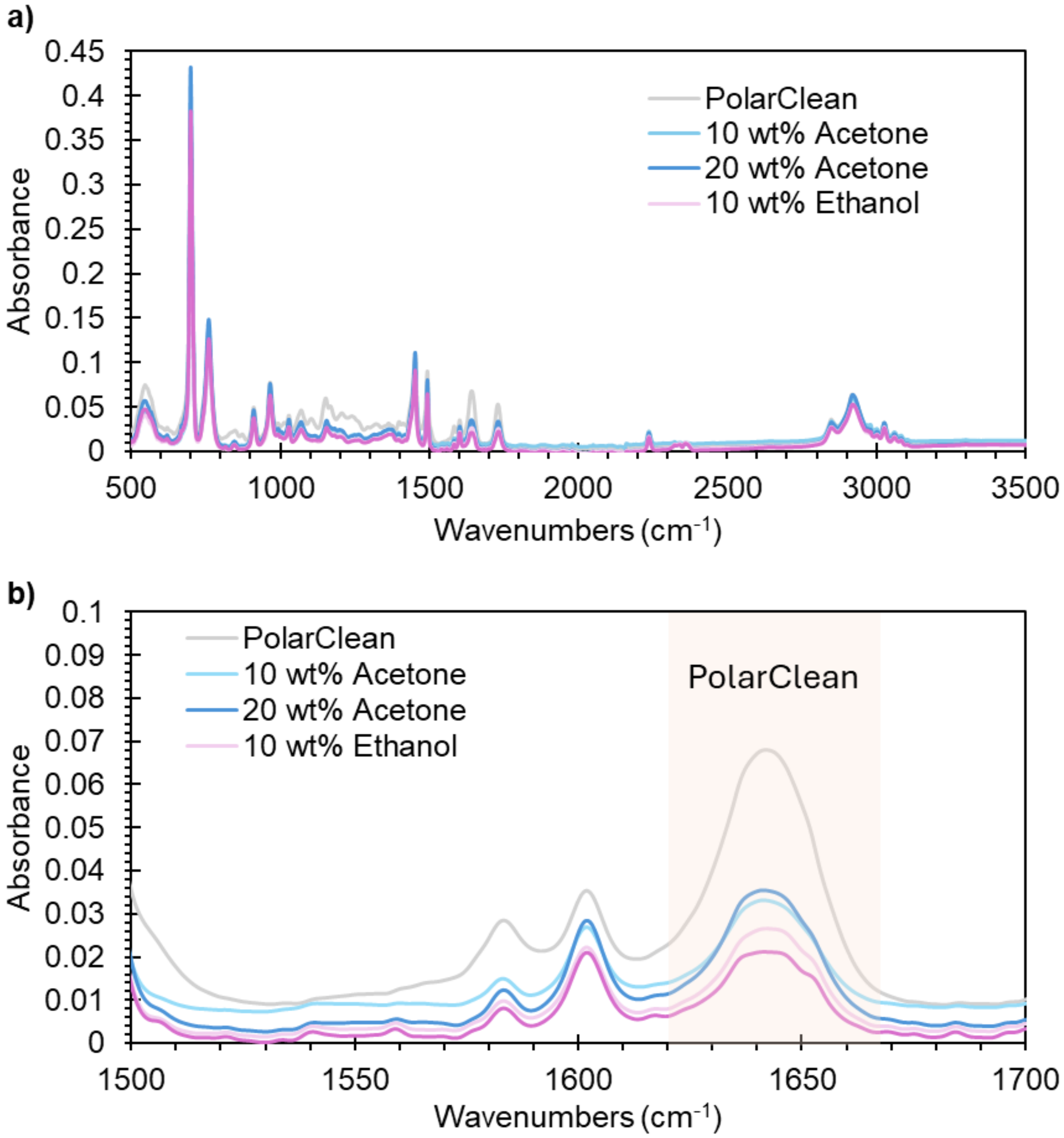

Figure S16. FTIR-ATR of 16wt% ABS membranes with pure PolarClean or diluents (10/20 wt% ethanol, 10/20wt% acetone) a) the full spectra b) zoomed in spectra on the region of a unique peak corresponding to PolarClean residual in the membrane indicating that all membranes have some PolarClean left over in the structure.

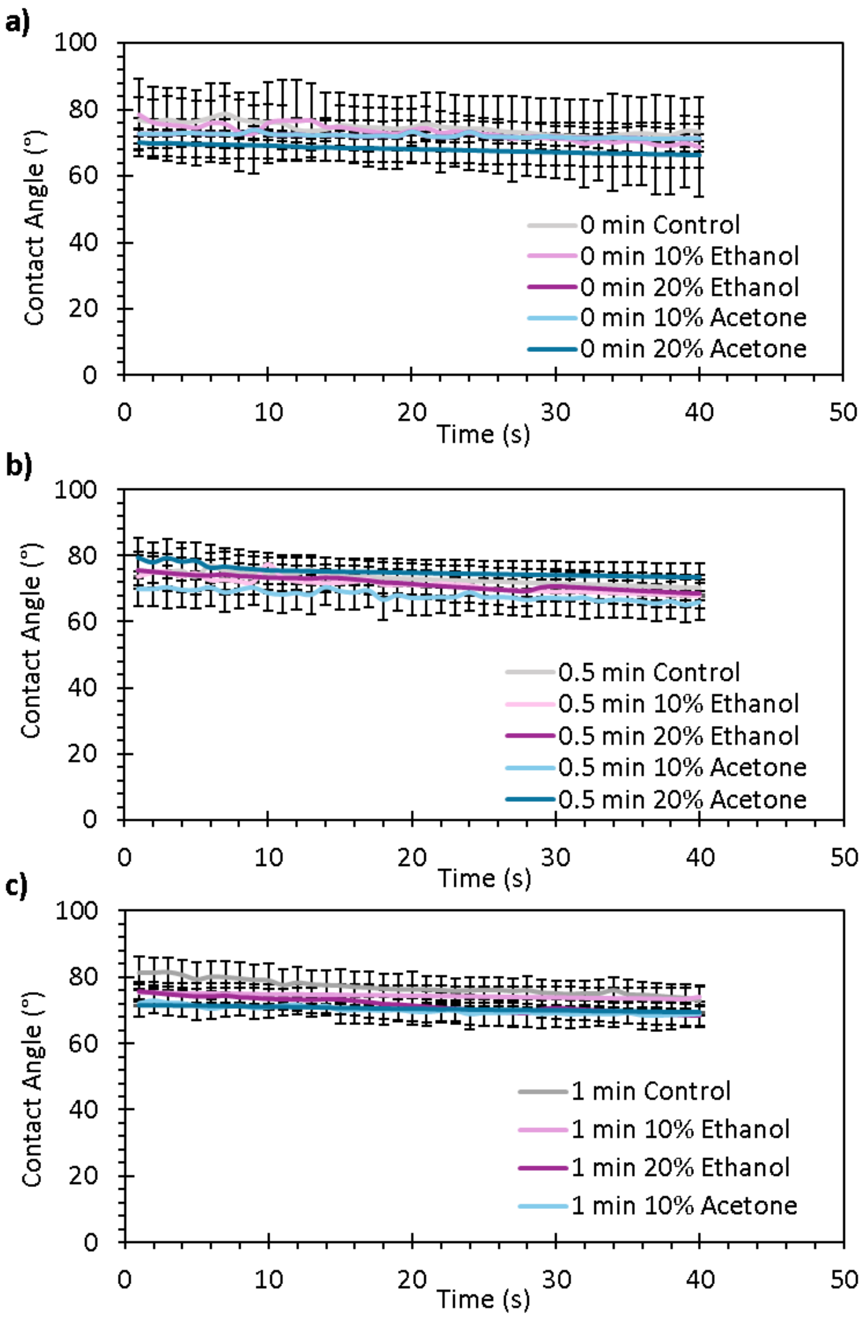

Figure S17. Contact angle of ABS membranes measured using a sessile drop analysis. a) Contact angle of membranes with zero minutes of solvent evaporation time. b) Contact angle of membranes with 0.5 minutes of solvent evaporation time. c) Contact angle of membranes with 1 minute of solvent evaporation time. The contact angle of all membranes was below 90°, signifying good wettability and thus, hydrophilic nature of the membrane surface. 20% Acetone with evaporation times of 0 and 1 minute had the lowest contact angle. However, all membranes had contact angles comparable or below that of the control membranes. .

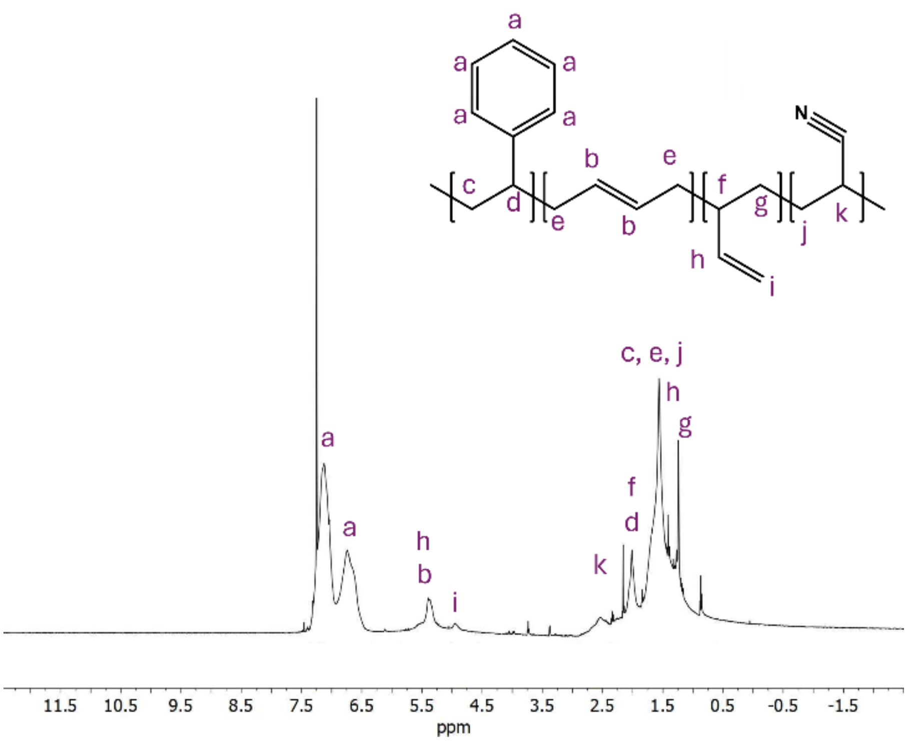

Figure S18. ABS 1H NMR spectra showing a composition of 48% poly(styrene), 14% poly(butadiene), 38% poly(acrylonitrile) performed in CDCl3.

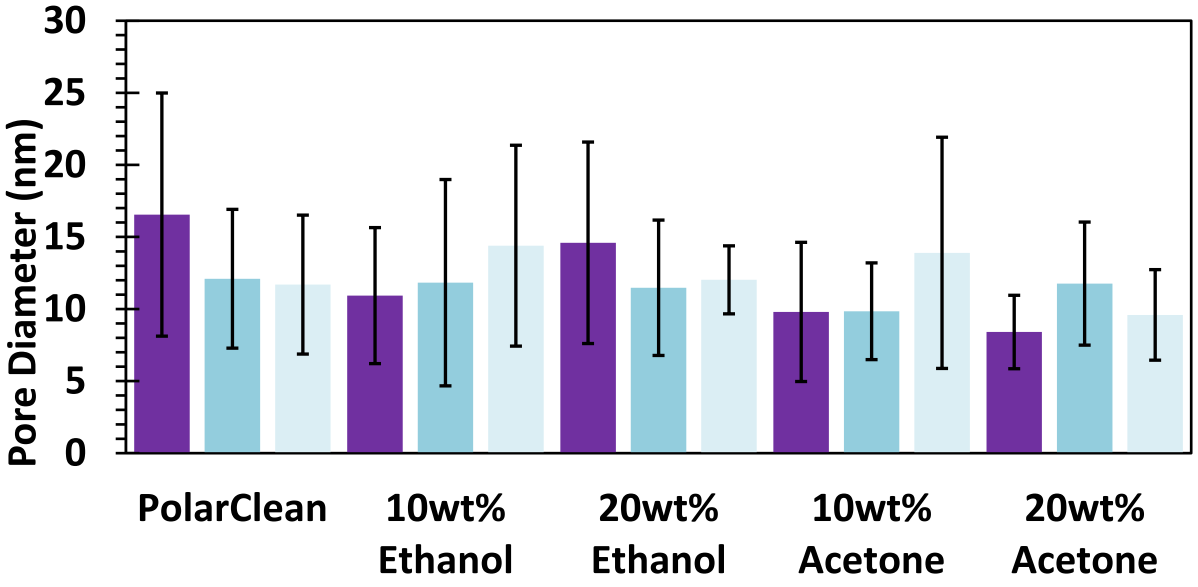

Figure S19. Average pore size of the membrane. SEM images of top surfaces, previously coated with 3 nm of Pt to increase conductivity, were analyzed via MATLAB and ImageJ to detect and quantity number of pores. The pores less than 5 nm in diameter were excluded.

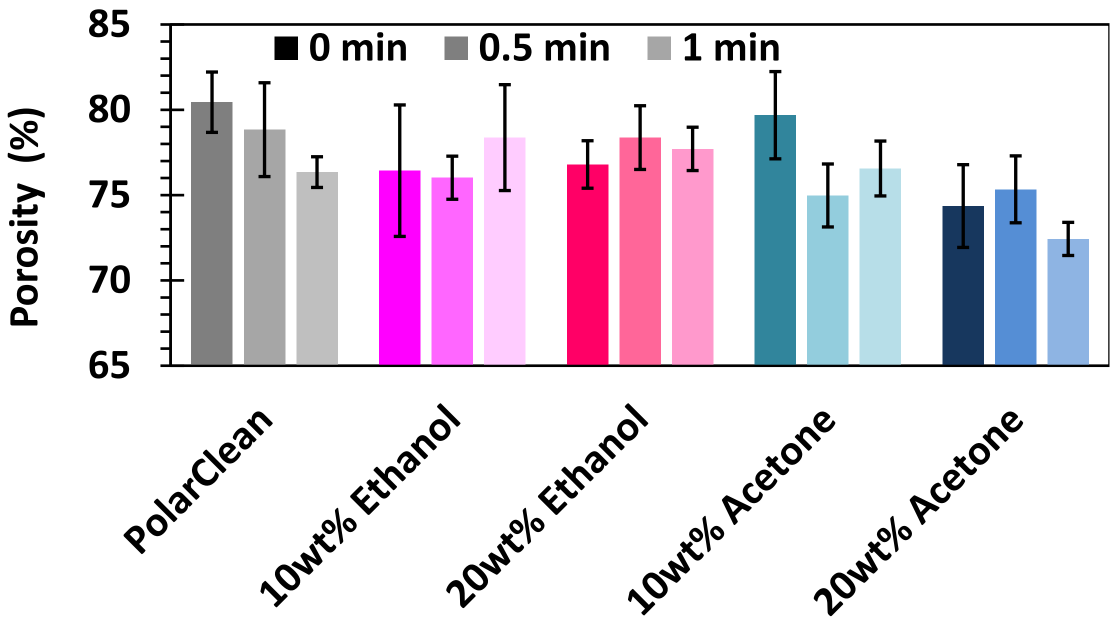

Figure S20. The impact of the 1 min evaporation time on the ABS membrane porosity. The porosity dropped for all membranes except for ethanol containing solutions which undergo the spinodal decomposition.

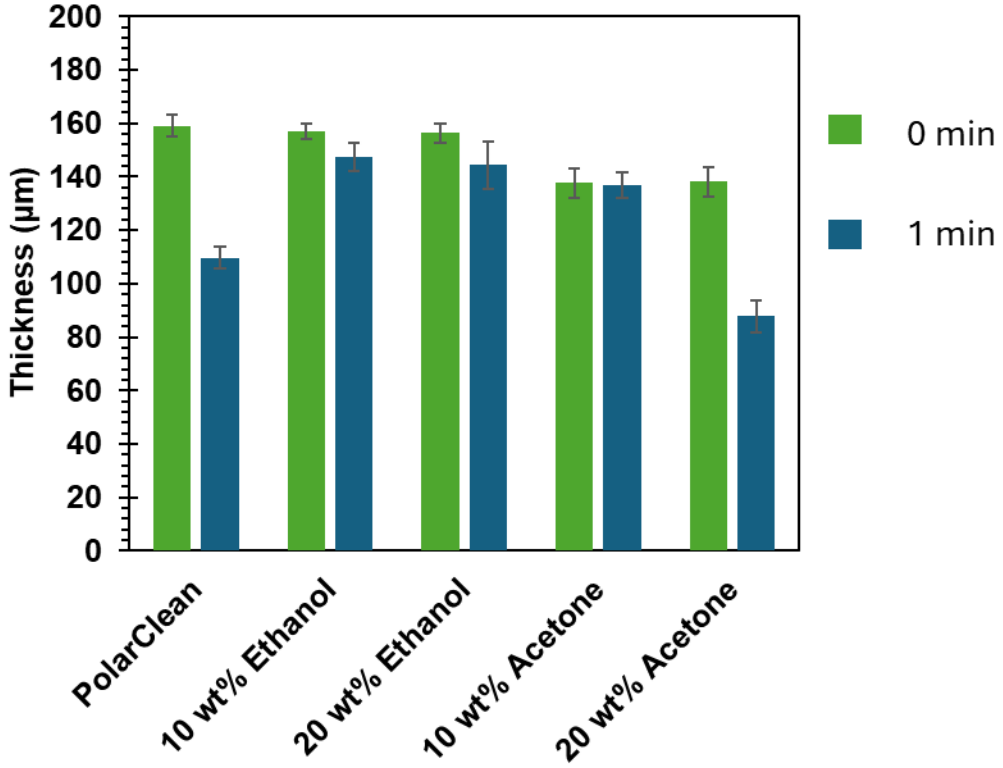

Figure S21. The impact of the 1 min evaporation time on the ABS membrane thickness; membrane thickness decreased after one minute of evaporation, with the greatest drop in 20 wt% acetone due to solvent volatility and early surface solidification. In the PolarClean-only membrane, humidity likely triggered early phase separation, limiting swelling during immersion and resulting in a thinner structure.

Table S3. Comparative Mechanical Properties of Common Flat Membrane Polymers from the Literature.

Polymer Membrane	Tensile Strength (MPa)	Elongation at Break (%)	References
**Acrylonitrile butadiene styrene (ABS)**	2-4	2–7	This study
**Polyethersulfone (PES)**	2-6	7	[1-3]
**Polysulfones (PSf)**	0.23-2	2.5-13	[4, 5]
**Polyvinylidene Fluoride (PVDF)**	~6 to ~11	20-200	[6]

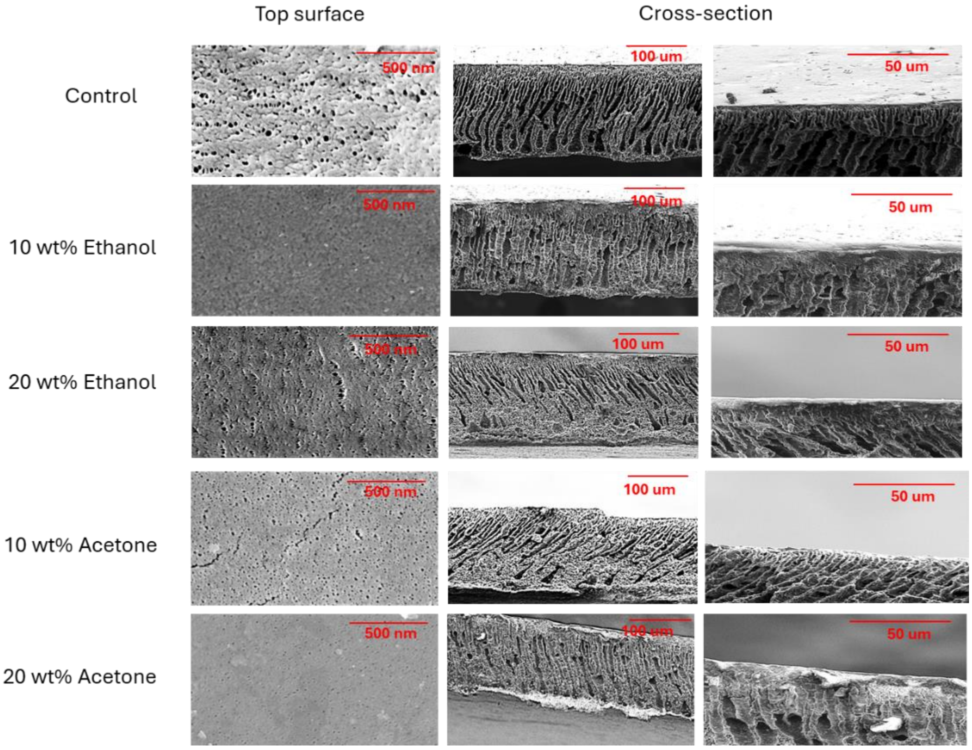

Figure S22. The top surface and cross section SEMs of the cast membranes with zero evaporation time.

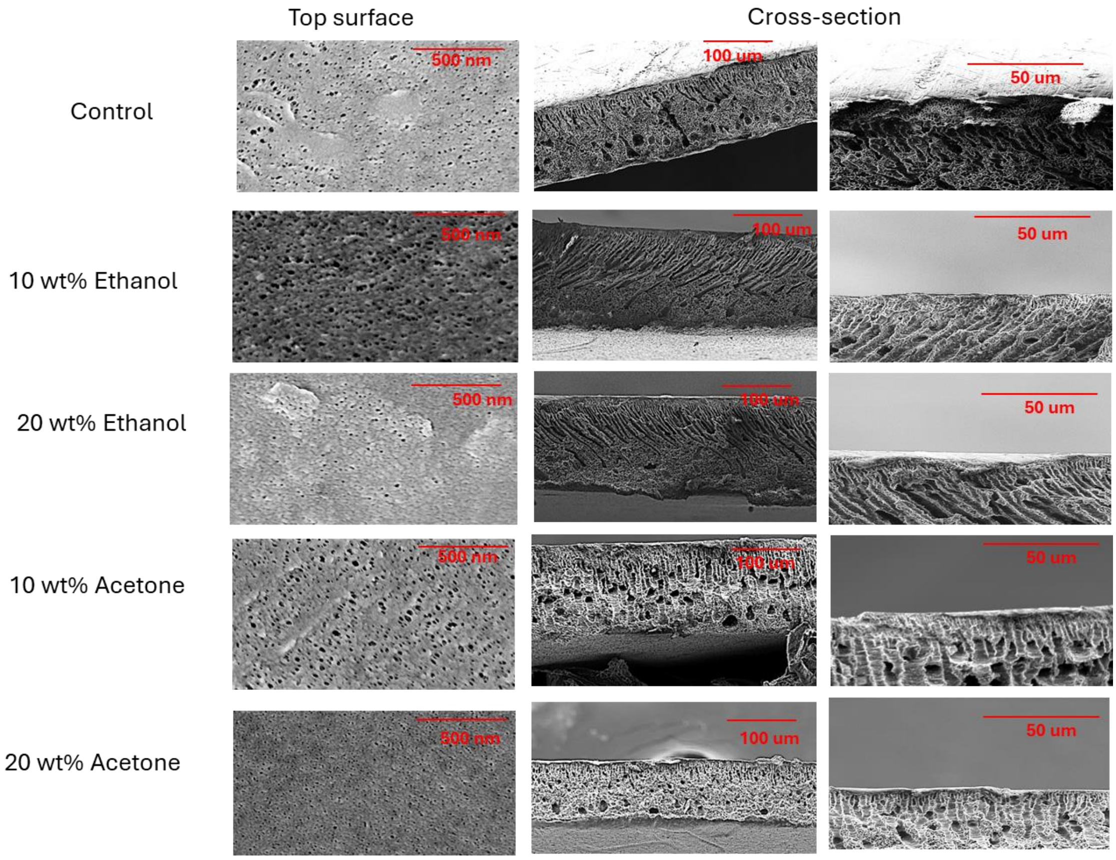

Figure S23. The top surface and cross section SEMs of the cast membranes with one min evaporation time.p

References

1. Jenkins, D., et al., *PoreScript: Semi-automated pore size algorithm for scaffold characterization.* Bioact Mater, 2022. **13**: p. 1-8.
2. Abbott, S. and C.M. Hansen, *Hansen solubility parameters in practice*. 2008: Hansen-Solubility.
3. Hansen, C.M., *Hansen solubility parameters: a user's handbook*. 2007: CRC press.
4. Peng, P., et al., *Relationship between Hansen Solubility Parameters of ABS and its Homopolymer Components of PAN, PB, and PS.* Journal of Macromolecular Science, Part B, 2010. **49**(5): p. 864-869.
5. Niskanen, J. and H. Tenhu, *How to manipulate the upper critical solution temperature (UCST)?* Polymer Chemistry, 2017. **8**(1): p. 220-232.
6. Nan, Y., et al., *Synergistic Approaches in the Design and Applications of UCST Polymers.* Macromol Rapid Commun, 2023. **44**(23): p. e2300261.
7. Zhang, Q. and R. Hoogenboom, *Polymers with upper critical solution temperature behavior in alcohol/water solvent mixtures.* Progress in Polymer Science, 2015. **48**: p. 122-142.
